# FOXA1 and adaptive response determinants to HER2 targeted therapy in TBCRC 036

**DOI:** 10.1038/s41523-021-00258-0

**Published:** 2021-05-12

**Authors:** Steven P. Angus, Timothy J. Stuhlmiller, Gaurav Mehta, Samantha M. Bevill, Daniel R. Goulet, J. Felix Olivares-Quintero, Michael P. East, Maki Tanioka, Jon S. Zawistowski, Darshan Singh, Noah Sciaky, Xin Chen, Xiaping He, Naim U. Rashid, Lynn Chollet-Hinton, Cheng Fan, Matthew G. Soloway, Patricia A. Spears, Stuart Jefferys, Joel S. Parker, Kristalyn K. Gallagher, Andres Forero-Torres, Ian E. Krop, Alastair M. Thompson, Rashmi Murthy, Michael L. Gatza, Charles M. Perou, H. Shelton Earp, Lisa A. Carey, Gary L. Johnson

**Affiliations:** 1 Department of Pharmacology, UNC Chapel Hill, Chapel Hill, NC USA; 2 Department of Radiation Oncology, Rutgers Cancer Institute of New Jersey, New Brunswick, NJ USA; 3 UNC Lineberger Comprehensive Cancer Center, UNC Chapel Hill, Chapel Hill, NC USA; 4 Department of Genetics, UNC Chapel Hill, Chapel Hill, NC USA; 5 Department of Biostatistics, UNC Chapel Hill, Chapel Hill, NC USA; 6 Department of Surgery, UNC Chapel Hill, Chapel Hill, NC USA; 7 University of Alabama-Birmingham School of Medicine, Birmingham, AL USA; 8 Dana-Farber Cancer Institute, Harvard Medical School, Boston, MA USA; 9 Department of Breast Surgical Oncology, MD Anderson Cancer Center, Houston, TX USA; 10 Department of Breast Medical Oncology, MD Anderson Cancer Center, Houston, TX USA; 11 Department of Medicine, UNC Chapel Hill, Chapel Hill, NC USA; 12 Present Address: Department of Pediatrics, Indiana University School of Medicine, Indianapolis, IN USA; 13 Present Address: Department of Pharmacology & Toxicology, Indiana University School of Medicine, Indianapolis, IN USA; 14 Present Address: Massachusetts General Hospital, Cambridge, MA USA; 15 Present Address: Koch Institute, Massachusetts Institute of Technology, Boston, MA USA; 16 Present Address: Hyogo Cancer Center, Akashi, Japan; 17 Present Address: Seattle Genetics, Inc., Seattle, WA USA; 18 Present Address: Baylor College of Medicine, Houston, TX USA

**Keywords:** Breast cancer, Tumour heterogeneity

## Abstract

Inhibition of the HER2/ERBB2 receptor is a keystone to treating HER2-positive malignancies, particularly breast cancer, but a significant fraction of HER2-positive (HER2+) breast cancers recur or fail to respond. Anti-HER2 monoclonal antibodies, like trastuzumab or pertuzumab, and ATP active site inhibitors like lapatinib, commonly lack durability because of adaptive changes in the tumor leading to resistance. HER2+ cell line responses to inhibition with lapatinib were analyzed by RNAseq and ChIPseq to characterize transcriptional and epigenetic changes. Motif analysis of lapatinib-responsive genomic regions implicated the pioneer transcription factor FOXA1 as a mediator of adaptive responses. Lapatinib in combination with FOXA1 depletion led to dysregulation of enhancers, impaired adaptive upregulation of HER3, and decreased proliferation. HER2-directed therapy using clinically relevant drugs (trastuzumab with or without lapatinib or pertuzumab) in a 7-day clinical trial designed to examine early pharmacodynamic response to antibody-based anti-HER2 therapy showed reduced FOXA1 expression was coincident with decreased HER2 and HER3 levels, decreased proliferation gene signatures, and increased immune gene signatures. This highlights the importance of the immune response to anti-HER2 antibodies and suggests that inhibiting FOXA1-mediated adaptive responses in combination with HER2 targeting is a potential therapeutic strategy.

## Introduction

Amplification and overexpression of v-erb-b2 erythroblastic leukemia viral oncogene homolog 2 (ERBB2, also known as HER2) occurs in up to 20% of breast cancers and defines a distinct clinical and molecular subtype^1–3^. HER2 is a receptor tyrosine kinase belonging to the epidermal growth factor receptor (EGFR) family that also includes HER3 (ERBB3) and HER4 (ERBB4)^4^. Consistent with the notion of oncogene addiction, treatment of HER2+ breast cancer has been significantly impacted by HER2-targeted therapies. Adding the anti-HER2 humanized monoclonal antibody trastuzumab, which targets the extracellular domain, to chemotherapy in early curable breast cancer decreases relapse and death by nearly 40%^5^. The development of additional HER2-targeted treatment options has expanded to include pertuzumab, a monoclonal antibody that blocks HER2 dimerization and significantly improves outcomes when added to trastuzumab-based therapy. Additionally, trastuzumab–emtansine and trastuzumab–deruxtecan, antibody–drug conjugates approved for adjuvant therapy (trastuzumab–emtansine) or later-line metastatic disease, and small-molecule tyrosine kinase inhibitors, such as tucatinib, lapatinib, and neratinib, which target EGFR and HER2^6^ are approved in the metastatic or adjuvant settings, usually with or after trastuzumab. In metastatic or early disease, up to 50% of patients respond to combinations of these HER2-targeting agents given with chemotherapy, however response rates to anti-HER2 therapy alone are low, and resistance remains a challenge. In the metastatic setting, continued HER2-targeting is standard across all lines of therapy, however, this is generally in combination with other drugs and virtually all ultimately experience disease progression and death.

Genomic and transcriptomic studies of HER2+ breast cancer have revealed substantial tumor and microenvironmental heterogeneity. While all intrinsic subtypes are represented, HER2+ breast cancer is primarily comprised of the HER2-Enriched, Luminal A, and Luminal B subtypes. Intrinsic subtype significantly impacts response to HER2-targeted therapy (reviewed in ref. ^7^). Evidence of immune activation, either by histologic presence of tumor infiltrating lymphocytes (TILS) or by immune gene expression, also significantly impacts response to HER2-directed therapy^8–10^.

In contrast to intrinsic resistance, characterized by initial failure to respond to HER2-targeted therapy, HER2+ tumors that respond to HER2-directed therapy often develop acquired resistance resulting in disease progression. Resistance has been attributed to numerous mechanisms, including the expression of a truncated HER2 (p95 HER2) that fails to bind trastuzumab, upregulation of downstream signaling via the PI3K/AKT pathway, and upregulation and increased signaling through alternate kinase pathways. The emergence of acquired resistance is due to the adaptive tumor responses to HER2 inhibition^11–13^. The adaptive response to targeted kinase inhibition involves disruptions in feedback and feedforward signaling loops that lead to dramatic epigenetic and transcriptional changes within the tumor cells themselves, as well as stromal and immune cell populations^13,14^. A major contributor to anti-HER2 treatment in HER2+ disease is the resultant upregulation of HER3, the preferred heterodimerization partner of HER2^4,15^. HER2/HER3 heterodimers drive oncogenic signaling, predominantly via the PI3K/AKT pathway and the upregulation of HER3 and other kinases has been shown to overcome HER2 inhibition^11,12,16–18^.

Adaptive responses to kinase inhibition have been observed in cell lines, animal models, and patient populations at early time points following treatment^14,19^. Adaptive kinome responses to MEK1/2 inhibition observed in triple-negative breast cancer patients after 7 days was shown in preclinical models to be dependent on transcriptional changes resulting from epigenetic genome-wide remodeling of the enhancer landscape^20^. The transcriptional output of adaptive response genes is governed by promoter and enhancer elements, including a number of super-enhancers (SEs), which are clusters of enhancers that impart cell identity^21^. A small number of master transcription factors (TFs) bind to SEs and define a given cell type or lineage. It has been hypothesized that these transcriptional regulatory programs may constitute cancer cell dependencies that could be targeted therapeutically^22^.

Studies from our laboratory have shown that the tumor cell-intrinsic adaptive response to lapatinib in HER2+ models resulted in the upregulation of HER3 and additional kinases, each contributing to resistance. The combination of lapatinib with the bromodomain and extra-terminal domain (BET) inhibitor, JQ1, was capable of blocking the transcription of kinases involved in the adaptive response and was more durable than lapatinib alone in growth suppression^12^. The BET family member BRD4 is an epigenetic reader, recognizing acetylated lysine residues on histones or other proteins and binding to key gene promoters and enhancers^21,23,24^. Here, we extend our findings by determining the global alterations in gene enhancers and transcriptional changes to identify factors involved in the adaptive response to HER2 inhibition. In parallel, we analyzed the in vivo human adaptive molecular responses to HER2 targeting in a window-of-opportunity clinical trial using both RNAseq and a chemical proteomics method (MIB/MS) to assess the functional kinome.

Integrative analysis of adaptive response SEs and mRNA expression identified a shared motif bound by FOXA1, a pioneer factor for estrogen receptor (ER). We show that FOXA1 is essential for the proliferation of HER2+/ER− cells and for the lapatinib-dependent upregulation of numerous genes, including HER3. In a window-of-opportunity trial using FDA-approved HER2-targeting drugs, a subset of patients exhibited a striking molecular response after 7 days of therapy. The expression of FOXA1 was drastically reduced in this subset, coincident with decreased HER2 and HER3 expression, decreased proliferation pathway signatures, and increased immune signatures. The strongest molecular responsive samples had higher HER2 amplicon pathway scores at baseline. Our studies implicate FOXA1 as a mediator of dynamic and adaptive transcriptional responses to HER2-targeted therapy.

## Results

### Epigenetic dysregulation induced by concurrent inhibition of HER2 and BET bromodomains

**Fig. 1:**
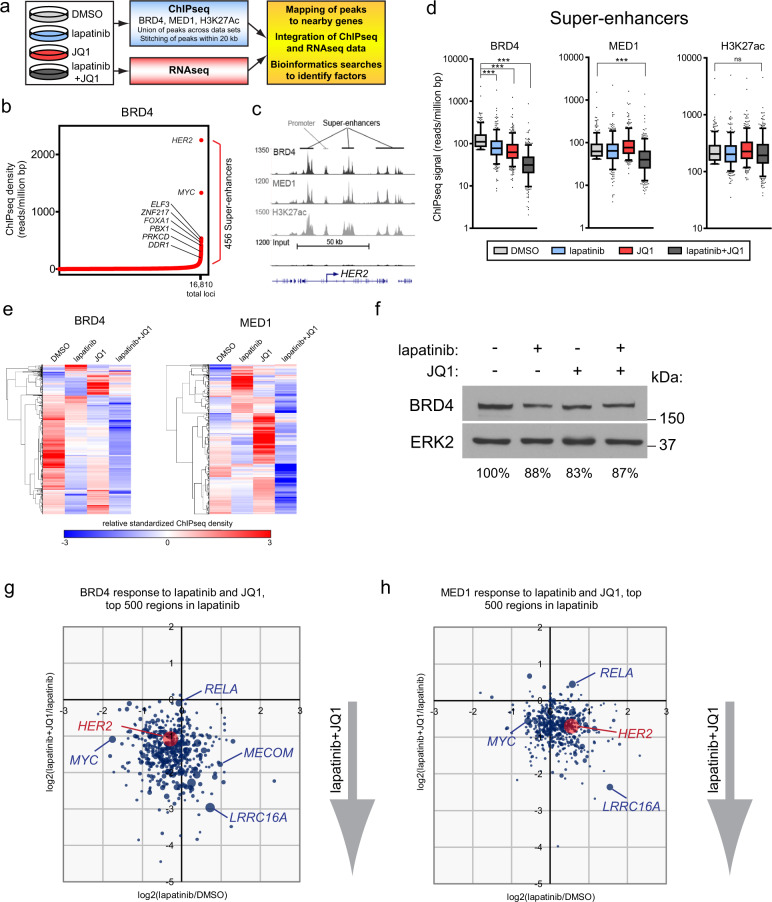
Combined inhibition of HER2 and BET bromodomains induces broad epigenetic dysregulation. **a** Experimental strategy to identify epigenetic and transcriptional adaptive responses to HER2 inhibition in cell line models using 300 nM lapatinib and 300 nM JQ1 for 24 h, alone or in combination. **b** ChIPseq analysis of the HER2+ breast cancer cell line, SKBR-3, was performed to identify BRD4 binding sites. Super-enhancers (456 regions) were identified as regions above the inflection point of increasing BRD4 ChIPseq density. **c** Multiple SEs identified by high density of BRD4, MED1, and H3K27Ac are found flanking the *HER2* locus. **d** BRD4 and MED1 binding to SE domains is significantly reduced by the combination of lapatinib and JQ1, but H3K27Ac is unaffected. Box plots: median, upper/lower quartile, and 5–95 percentile. Unpaired *t*-test, ***= *P* < 0.0001. **e** Unsupervised hierarchical clustering of all genomic loci bound by BRD4 and MED1. Lapatinib and JQ1 each increase BRD4 and MED1 binding to discrete regions but the combination of drugs results in a loss of binding at the majority of loci. **f** Total protein levels of BRD4 are largely unaffected by lapatinib and JQ1 as determined by immunoblotting of treated SKBR-3 cells. ERK2 was used as a loading control. **g**, **h** The combination of lapatinib and JQ1 cooperatively reduce BRD4 and MED1 binding to *HER2* and *MYC*. The top 500 regions of highest ChIPseq density in response to lapatinib are shown for **g** BRD4 and **h** MED1. The log_2_ fold change in density in response to lapatinib vs. DMSO is plotted on the *x*-axis. The log_2_ fold change of lapatatinib + JQ1 vs. lapatinib alone is plotted on the *y*-axis. Dot size is relative to ChIP density in lapatinib.

Our prior studies demonstrated the adaptive response and acquired resistance patterns of the kinome to lapatinib treatment could be blocked by JQ1^12^. ChIP-PCR revealed that JQ1 alone could disrupt BRD4 association with the *HER2* and *HER3* gene promoters. Global analysis using ChIPseq revealed broad epigenetic alterations induced by HER2 targeting (Fig. 1a). BRD4 is a critical mediator of cell identity controlling enhancer architecture in response to perturbations such as kinase inhibitors. Although the strict classification of SEs can vary, a unifying feature of SEs is the heightened enrichment of BRD4, mediator subunit MED1, binding of master transcription factors, and acetylation of lysine 27 of histone H3 (H3K27ac)^22,25^. Given our interest in identifying the kinases potentially impacted by the BET bromodomain inhibitor JQ1, we focused on the regions with highest BRD4 ChIPseq density. Analysis of BRD4 density by ChIPseq was used to identify SEs and their classification based on their location in the genome (enhancer, promoter, gene body intron, gene body exon, 3 prime, or other, see “Methods”) in an unbiased fashion in two HER2+ breast cancer cell lines, SKBR-3 (ER−/PR−) and BT474.m1 (ER+/PR+) (Fig. 1b, Supplementary Fig. 1a and Supplementary Data 1 and 2). Ranking BRD4 enrichment by ChIPseq density can be used to classify SEs regulating genes critical for cell identity^21^. The majority of identified BRD4 peaks (74%) were classified as promoter or enhancer regions based on genomic location and virtually all of the identified SEs (95%) in SKBR-3 cells were classified as promoter or enhancer regions. BRD4 was highly enriched proximal to *HER2* and the essential proto-oncogene *MYC*. Other BRD4-enriched regions identified were proximal to TFs, including *ELF3*, *ZNF217*, *FOXA1*, and *PBX1*, each with recognized function in epithelial cell identity and in breast cancer^26–29^. BRD4 enrichment at the estrogen receptor alpha gene, *ESR1*, was identified only in the HER2+/ER+ BT474.m1 cells (Supplementary Fig. 1a). Shared BRD4 enrichment and SE classification between the two cell lines was observed at *HER2*, *FOXA1*, and *PBX1* (Fig. 1b and Supplementary Fig. 1a). FOXA1 and PBX1 have been characterized as pioneer factors for ER, important for increasing chromatin accessibility for ER and other TFs^28,29^. As expected, the enrichment of BRD4 near the *HER2* locus coincided with mediator subunit MED1 chromatin association and the presence of the active enhancer mark, H3K27Ac (Fig. 1c).

We previously demonstrated that JQ1 could prevent the upregulation of numerous kinases contributing to lapatinib response and resistance^12^. Analysis of enrichment in SKBR-3 and BT474.m1 cells revealed that BRD4 and MED1 chromatin association was significantly disrupted by the combination of lapatinib and JQ1 at 24 h following treatment when compared to DMSO (Fig. 1d and Supplementary Fig. 1b). Unsupervised hierarchical clustering of BRD4 and MED1 relative ChIPseq density at all genomic loci confirmed that the combination of lapatinib and JQ1 drastically disrupted their chromatin association (Fig. 1e). The effects on ChIPseq density of BRD4 and MED1, were not due to a reduction in total BRD4 protein levels as determined by immunoblotting (Fig. 1f). The potent effect on BRD4 and MED1 chromatin association was consistent with 24 h exposure to targeting BET proteins with JQ1.

Given the effectiveness of JQ1 in blocking the adaptive response to lapatinib, we examined the impact of JQ1 on BRD4 and MED1 chromatin binding at regions most affected by lapatinib alone (Fig. 1g and h, dot sizes proportional to ChIPseq density). The top 500 regions with the highest BRD4 enrichment in response to lapatinib were predominantly classified as promoter or enhancer regions (95%, Supplementary Fig. 1e). The log_2_ fold change for lapatinib versus DMSO is plotted on the *x*-axis and identified regions that exhibited relative increased and decreased densities at the indicated loci. Strikingly, the comparison of lapatinib combined with JQ1 to lapatinib alone (plotted on the *y*-axis) revealed a dramatic effect on BRD4 and MED1 binding, notably at regions proximal to *HER2* and *MYC*. Similar findings were observed in the HER2+/ER+ line, BT474.m1—specifically at loci near *HER2*, *ESR1* (estrogen receptor 1), and *PGR* (progesterone receptor) (Supplementary Fig. 1c). Collectively, these data indicated that lapatinib and JQ1 cooperatively disrupt BRD4 and MED1 chromatin binding at a majority of genomic loci, consistent with their capacity to elicit potent effects on adaptive responses and suppress tumor cell growth^12^.

### Regions with increased ChIPseq density and proximal mRNA expression in response to lapatinib are enriched for a FOXA1-binding motif

**Fig. 2:**
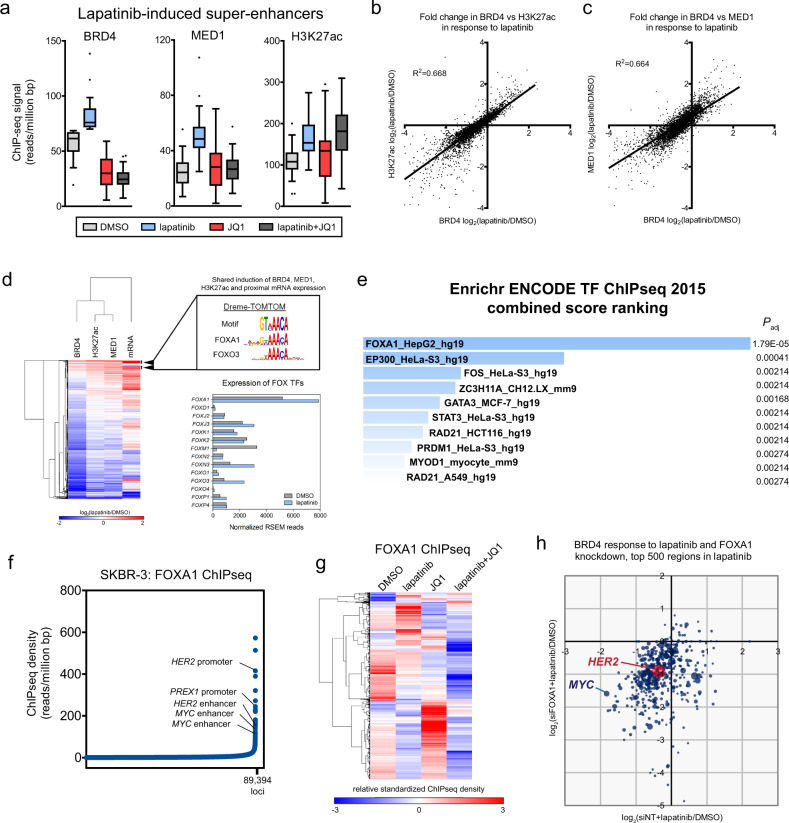
Lapatinib-induced ChIPseq and RNAseq changes are associated with FOX family transcription factors. **a** Lapatinib induces 30 SEs, as determined by BRD4 ChIPseq density. Lapatinib in combination with JQ1 suppresses BRD4 binding in all regions, MED1 in all but one region, but does not affect H3K27Ac. Box plots: median, upper/lower quartile, and 1.5 IQR. **b**, **c** Lapatinib-mediated changes are strongly correlated with changes in H3K27Ac and MED1. **d** The log_2_ fold change binding in response to lapatinib for regions as in (**a**) and the log_2_ fold change in mRNA expression for the proximal genes as determined by RNAseq was used for unsupervised hierarchical clustering. Genes included based on ChIPseq density displayed increased chromatin association within 200 kb of transcription start site. Genes with shared induction in all instances (log_2_ fold change ≥1) were used as input for Dreme-TOMTOM analysis to identify enriched binding motifs. A single motif identified, GT(A/C)AACA, recognized by FOXA1 and FOXO3. Normalized RNAseq reads are shown for the FOX family of transcription factors in SKBR-3 cells after 48 h treatment with DMSO or 300 nM lapatinib. **e** Genes from (**d**) were used to query Enrichr. ENCODE TF ChIPseq data identified FOXA1 and p300 as potential factors. **f** ChIPseq analysis of FOXA1 binding in SKBR-3 cells with the regions of highest density annotated by proximal gene. **g** Unsupervised hierarchical clustering of FOXA1 ChIPseq density in SKBR-3 cells treated with DMSO, 300 nM lapatinib, and 300 nM JQ1, alone or in combination, at all genomic loci. Lapatinib and JQ1 each result in regions of increased FOXA1 chromatin binding. The combination of lapatinib and JQ1 results in broad disruption of FOXA1. **h** SKBR-3 cells were treated with siRNA pools, nontargeting (NT) control or FOXA1, prior to DMSO or 300 nM lapatinib treatment for 24 h and subsequent BRD4 ChIPseq analysis. The top 500 regions in lapatinib are plotted. Log_2_ fold change of lapatinib vs. DMSO with a control siRNA pool are plotted on the x-axis. Log_2_ fold changes of lapatinib vs. DMSO with FOXA1 knockdown are plotted on the y-axis. Dots are scaled relative to their lapatinib-induced ChIPseq density.

While the combination of lapatinib and JQ1 resulted in major dysregulation of enhancers, we sought to better understand the epigenetic changes occurring in response to lapatinib alone. We identified SEs formed in response to lapatinib in SKBR-3 and BT474.m1 cells (Fig. 2a and Supplementary Fig. 2a). Approximately 30 SEs were formed in SKBR-3 cells based on BRD4 association following 24 h treatment with lapatinib. BRD4 and MED1 ChIPseq density at these regions was suppressed by the addition of JQ1. H3K27Ac at these lapatinib-induced regions was not affected by the addition of JQ1 at 24 h following treatment. Overall, the global changes in BRD4 in response to lapatinib correlated with H3K27Ac and MED1 ChIPseq density (Fig. 2b and c).

While we had previously identified a heterogenous kinome reprogramming response to lapatinib, we did not observe de novo SE formation at these kinase genes (Supplementary Fig. 2b and c). For example, SKBR-3 cells undergo increased expression of *HER3* and *DDR1* in response to lapatinib, but the ChIPseq density of BRD4, MED1, and H3K27Ac did not increase at these genes (Supplementary Fig. 2b and c). We confirmed increased SE markers for some genes in response to lapatinib, such as *PGR* in the ER+ BT474.m1 cell line (Supplementary Fig. 2d).

Since the SEs formed in response to lapatinib did not provide a clear explanation for the adaptive kinome response, we employed integrative analysis of the ChIPseq data with mRNA expression data to identify underlying adaptive response factors. Lapatinib-induced changes (log_2_ fold change) in ChIPseq density for BRD4, MED1, and H3K27Ac (not only the regions designated as SEs) and mRNA expression changes from RNAseq were utilized for unsupervised hierarchical clustering, revealing that small subsets of genes appeared to respond in similar fashion (Fig. 2d). In order to define functional genomic elements, we identified underlying genetic sequences that exhibited at least two-fold increases in ChIPseq density and mRNA expression of proximal genes in response to lapatinib. Dreme-TOMTOM analysis of these sequences identified a single motif, GT(A/C)AACA, associated with the FOX TFs, FOXA1, and FOXO3 (Fig. 2d). Analysis of the normalized RNAseq read counts for the FOX family of TFs in SKBR-3 cells indicated that FOXA1 is the most highly expressed member. Using Enrichr to query the list of genes having shared induction of local ChIPseq density and mRNA expression, we identified a FOXA1 ChIPseq data set from HepG2 cells with the highest combined score from the ENCODE ChIPseq 2015 data (Fig. 2e). Taken together, our analysis strongly suggested that FOXA1 was orchestrating the lapatinib-induced adaptive response.

We performed ChIPseq analysis of FOXA1 in the ER- SKBR-3 cell line (Fig. 2f and Supplementary Data 1). The highest FOXA1 enrichment mapped to discrete gene regulatory regions including the *HER2* promoter and enhancer as well as enhancers for *MYC*. Unsupervised hierarchical clustering of FOXA1 ChIPseq density revealed the near global effect on FOXA1 chromatin association in response to combined lapatinib and JQ1 treatment (Fig. 2g). Lapatinib treatment combined with FOXA1 depletion by RNAi strongly reduced the association of BRD4 with *HER2* and *MYC* enhancers when compared to lapatinib and a non-targeting control siRNA (Fig. 2h and Supplementary Data 3). HER2 targeting combined with JQ1 significantly reduced FOXA1 chromatin binding genome-wide and similarly, targeting HER2 and FOXA1 disrupted enhancers at essential genes.

**Fig. 3:**
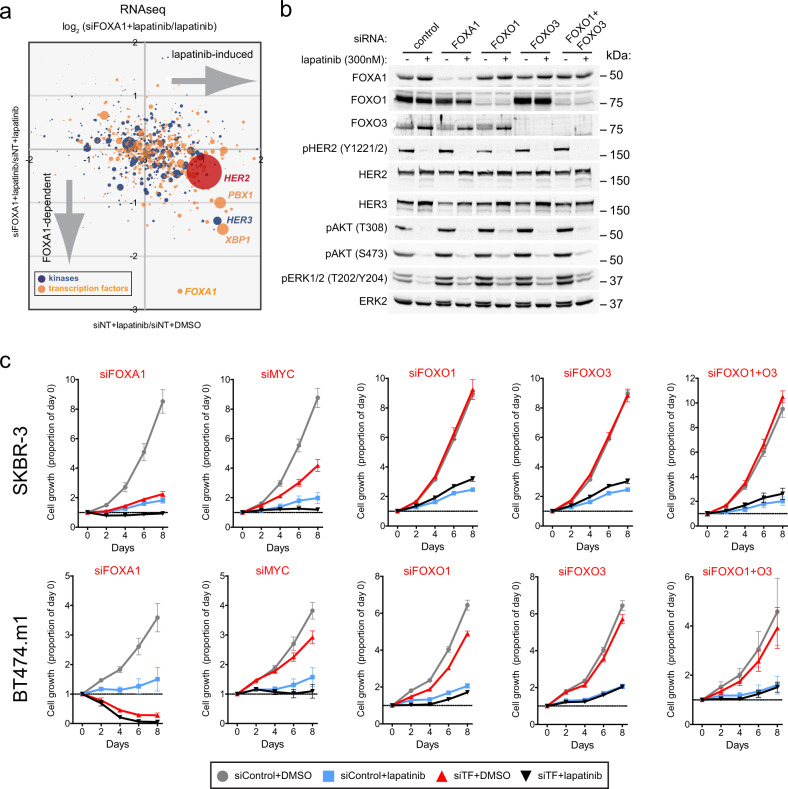
FOXA1 is critical for the HER2 targeting response and for proliferation in HER2+ cell lines. **a** RNAseq was performed on SKBR-3 cells treated with DMSO or 300 nM lapatinib for 24 h after treatment with a non-targeting (NT) siRNA pool or *FOXA1* siRNA pool. The log_2_ fold changes in response to lapatinib are plotted as the NT siRNA effect (*x*-axis) versus *FOXA1* siRNA effect (*y*-axis) for kinases (blue) and transcription factors (orange). *HER2* is indicated in red. Dots sizes are scaled relative to their expression level. **b** SKBR-3 cells were treated with siRNA as in (**a**), then DMSO or 300 nM lapatinib for 24 h, harvested and subjected to immunoblotting to detect the indicated proteins. ERK2 was used as a loading control. **c** SKBR-3 and BT474.m1 cells were transfected with a NT siRNA pool or siRNA pools targeting the indicated transcription factor (TF) and then treated with DMSO or 300 nM lapatinib for 8 days. Cell growth was quantified every two days and plotted as the proportion of day 0 cell count. Data are plotted as mean ± s.d. for six replicates.

To determine whether FOXA1 was required for adaptive transcriptional responses to HER2 targeting, gene expression in response to lapatinib was analyzed by RNAseq with non-targeting control or FOXA1 siRNA (Fig. 3a and Supplementary Data 4). The log_2_ fold changes for kinases (blue) and TFs (orange) are shown with the dot size proportional to the gene expression level in the presence of lapatinib. FOXA1 depletion most notably reduced the lapatinib-dependent induction of *HER2, HER3, PBX1,* and *XBP1* (Fig. 3a). The expression of FOXA1 is also induced by lapatinib, but the effects of siRNA targeting were more readily observed. Closer inspection of the *HER3* locus identified two intronic enhancers displaying enhanced recruitment of FOXA1 following lapatinib treatment (Supplementary Fig. 3). These regions are blocked by JQ1 treatment and the intron 1 enhancer has been previously implicated in *HER3* regulation by specific TFs^30–33^. By immunoblotting, we confirmed the depletion of FOXA1 and the lapatinib-dependent induction of HER3 and compared the effects of FOXO1 and FOXO3 depletion by RNAi, singly or in combination. We did observe a lapatinib-induced increase in FOXO3 protein levels. After FOXO3 RNAi treatment and 24 h lapatinib exposure, there was no observable effect on phospho-HER2, phospho-AKT, or phospho-ERK levels compared to lapatinib and control RNAi (Fig. 3b).

To directly assess the requirement of FOXA1 for proliferation and response to HER2 targeting, we performed cell proliferation assays in the SKBR-3 and BT474.m1 cell lines (Fig. 3c). While both cell lines are strongly responsive to lapatinib (compare blue to gray lines), the effect of targeting FOXA1 alone by siRNA was comparable to 300 nM lapatinib treatment in SKBR-3 cells and resulted in the loss of BT474.m1 cells. In the Cancer Dependency Map (depmap.org), the top FOXA1 co-dependencies from the CRISPR screen data (AVANA) by Pearson correlation are SPDEF followed by ESR1 (estrogen receptor alpha) and TRPS1, underscoring the critical interplay of FOXA1 and ESR1^34^. In comparison, siRNA knockdown of MYC inhibited proliferation in both ER+ BT474.m12 and ER− SKBR-3 cells, but less dramatically than HER2 targeting with lapatinib. Since our survey of super-enhancers had identified a TF binding motif that potentially involved FOXO1 or FOXO3, each gene was targeted by siRNA and in combination (Fig. 3c). FOXO1 and FOXO3 depletion did not impact cell growth when compared to FOXA1. These data confirmed that adaptive transcriptional responses to HER2 targeting, cell proliferation, and survival were dependent on FOXA1 expression.

### Distinct FOXA1 transcriptional response in a subset of patients receiving anti-HER2 therapy

**Fig. 4:**
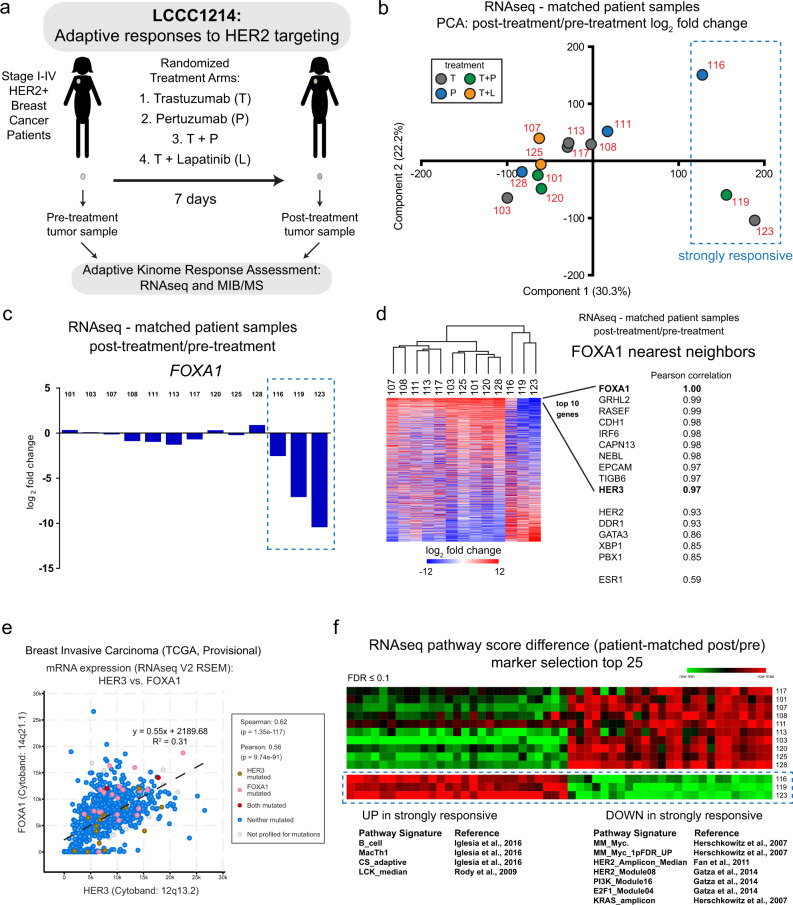
Distinctly responsive patient samples are marked by dramatic FOXA1 loss, HER3 and HER2 decreases and increased immune signatures. **a** Schematic overview of LCCC1214 (Clinical trials identifier NCT01875666) for the analysis of adaptive responses to HER2 targeted therapy. **b** Normalized RNAseq data from matched patient samples was used to generate log2 fold changes for expressed genes (post-treatment vs. pre-treatment). The fold change in expression was used for the principal component analysis (PCA). Labels indicate patient number and dot color indicates treatment arm. The blue dashed line indicates distinct matched samples (from patients 116, 119, and 123) determined to be strongly responsive based on their distinct expression changes. **c** Log_2_ fold change for *FOXA1* for matched patient samples is plotted. The blue dashed line indicates the strongly responsive sample pairs. **d** Log_2_ fold changes of the top 5000 differentially expressed genes for matched patient samples were used for unsupervised hierarchical clustering and nearest neighbor analysis was performed for the *FOXA1*. The top 10 genes by Pearson correlation are indicated, as well as select genes identified from HER2+ cell line SE analysis. **e** The breast invasive carcinoma (TCGA, provisional) data set was used to identify genes most significantly co-expressed with *HER3* expression in patient tumors. The top genes were *XBP1* (not shown) and *FOXA1*. The dashed line indicates regression line. Table contains legend for mutation status, if known. **f** Pathway analysis of gene expression data from matched patient samples was performed and the pathway scores differences was used for marker selection comparing the strongly responsive sample pairs (blue dashed line) to all other sample pairs. The top 25 pathways (increased and decreased) are shown (10% FDR cutoff). Representative significant pathways are listed with associated reference.

As our preclinical studies had underscored the importance of rapid adaptive responses to kinase inhibitors, we initiated a multi-center window-of-opportunity trial to illuminate these changes in HER2+ patients receiving standard-of-care treatment. Newly diagnosed stage I–IV HER2+ patients were enrolled into LCCC1214 (Clinical Trials Identifier #NCT01875666), randomized into four treatment groups and a pre-treatment/baseline fresh biopsy of the primary tumor was obtained. All treatment arms received a standard-of-care HER2-targeting antibody at standard clinical doses, singly or in combination: trastuzumab (T), pertuzumab (P), the combination of T + P, or T plus lapatinib (L) (Fig. 4a). Following seven days of therapy, patients returned for scheduled surgical procedure. At the time of surgery, post-treatment samples were obtained for analysis of adaptive molecular changes in response to HER2 targeting. Analysis included RNAseq to evaluate the transcriptional response to therapy and a chemical proteomics assay (MIB/MS) to enrich and quantify the functional kinome^19,20,35^.

For 13 patients, matched pre-treatment and post-treatment samples were collected while for 8 additional patients, only a pre-treatment or a post-treatment sample was obtained of sufficient quality for analysis. DESeq2 analysis of transcriptome data from all post-treatment samples versus all pre-treatment samples identified 185 genes that were significantly upregulated and associated with immune signatures, including PD1 and PD-L1 individual gene expression (Supplementary Fig. 4a, Supplementary Data 5, and Supplementary Data 6). This is consistent with a role for innate and adaptive immunity in response to HER2 antibody treatment^36^ and increasing recognition of the independent contribution of the immune microenvironment to response to HER2-directed drugs^8,37^. Other investigators have found that tumor infiltrating lymphocytes or increased immune gene expression 7–15 days after initiating trastuzumab or trastuzumab plus lapatinib are associated with higher pathologic complete response after several months of therapy^38,39^. Our data suggest specific immune activation patterns are part of the earliest response pattern, clearly detectable within 7 days of initiating therapy, and appear similar across antibody-based anti-HER2 treatments. The log_2_ fold change in expression for matched patient samples was used for principal component analysis of the transcriptome (Fig. 4b). Three matched pairs (from patients 116, 119, and 123) were notably distant from the other 10 patient pairs.

We also observed segregation of these sample pairs by unsupervised hierarchical clustering of the expressed kinome (Supplementary Fig. 4b). The three distinct sample pairs represented three different treatment arms (patient 116, P; 119, T + P, 123, T) so a specific treatment did not dictate their unique transcriptional response. Within these HER2+ tumors based on clinical criteria, all the molecular intrinsic subtypes were represented, however, there was no apparent relationship between subtype and the kinome expression changes induced by HER2-targeting drugs (Supplementary Fig. 4b).

Previous gene expression profiling studies have shown that clinical HER2+ samples include HER2-Enriched, Luminal A, Luminal B, Basal-like, and Normal-like subtypes and that the HER2-Enriched subtype has markedly higher clinical and pathologic responsiveness to standard anti-HER2 drugs^7,40^. Most pre- and post-treatment matched pairs that were less molecularly dynamic retained the same intrinsic subtype, with the exception of patient 125, whose sample subtype changed from Luminal A to Luminal B, patient 108 (Luminal B to Luminal A), and 113 (Luminal A to Normal-like). In the three strongly responsive paired samples, the intrinsic subtype of patient 116 changed from Basal-like to HER2-Enriched, patient 119 from HER2-Enriched to Luminal A, and patient 123 did not change and was classified as Luminal B at both time points. Collectively, these data indicated that intrinsic subtype could not explain these variations in short-term, drug-induced expression changes.

The three matched pairs of samples that underwent the strongest molecular response within 7 days did not exhibit significantly higher HER2 expression and were not exclusively HER2-Enriched intrinsic subtype at baseline. When the three pre-treatment samples were compared to all other pre-treatment samples they had significantly higher expression of *PSMD3*, a gene located within the *HER2* amplicon at 17q21.1 (Supplementary Fig. 4c and Supplementary Data 6). These pre-treatment samples also had higher HER2-amplicon gene expression module score when compared to the less responsive samples (Supplementary Fig. 4d and Supplementary Data 7). This observation supports other findings that increased *HER2* amplification or HER2 amplicon score may identify those tumors that are more HER2-addicted and more likely to exhibit a high degree of dynamic response^9^.

As our preclinical analysis had implicated FOXA1 in the adaptive response to HER2 inhibition, we assessed *FOXA1* expression levels in matched patient samples (Fig. 4c). The three strongly responsive samples identified by unsupervised approaches had the largest decreases in *FOXA1* in response to treatment. To identify genes behaving in a manner similar to *FOXA1*, we performed unsupervised hierarchical clustering of the log_2_ fold change of the top 5000 differentially expressed genes. Nearest neighbor analysis for the *FOXA1* gene response was performed to rank genes by Pearson correlation (Fig. 4d). The change in *FOXA1* strongly coincided with a number of epithelial marker genes (*GRHL2, CDH1*, and *EPCAM*) as well as *HER3*. Additionally, expression of *HER2*, *DDR1*, and the TFs *GATA3, XBP1*, and *PBX1* were also strongly correlated with *FOXA1*. To validate this observed correlation, we analyzed the genes correlated with *FOXA1* using TCGA provisional RNAseq data from the Breast Invasive Carcinoma samples using cbioportal.org^41,42^. The highest correlation with *FOXA1* was *ESR1* (ER), consistent with a critical role for FOXA1 as a pioneer factor for ER. However, when we used *HER3*, the most strongly correlated genes were *FOXA1* and *XBP1*, by Spearman correlation (Fig. 4e and data not shown).

Given the response of *FOXA1* in the three distinct matched sample pairs, we performed expression pathway analysis and marker selection to identify the most significantly altered pathway scores (as log_2_ fold change) in response to treatment (Fig. 4f). In addition to increases in a number of immune signatures, we observed decreases in HER2, HER2 amplicon, MYC, PI3K, KRAS, and E2F1 modules (10% FDR). These data revealed a distinct subset of patient sample pairs were characterized by rapid and dramatically reduced *FOXA1* expression coincident with *HER3* and *HER2* expression changes, as well as immune signature increases consistent with the trastuzumab and pertuzumab inducing an immune cell infiltration in these patient tumors and proliferative signature decreases. These strongly responsive samples showed higher HER2 amplicon pathway activity at baseline (pre-treatment).

### Distinctly responsive patient samples share discrete TF and functional kinome features

**Fig. 5:**
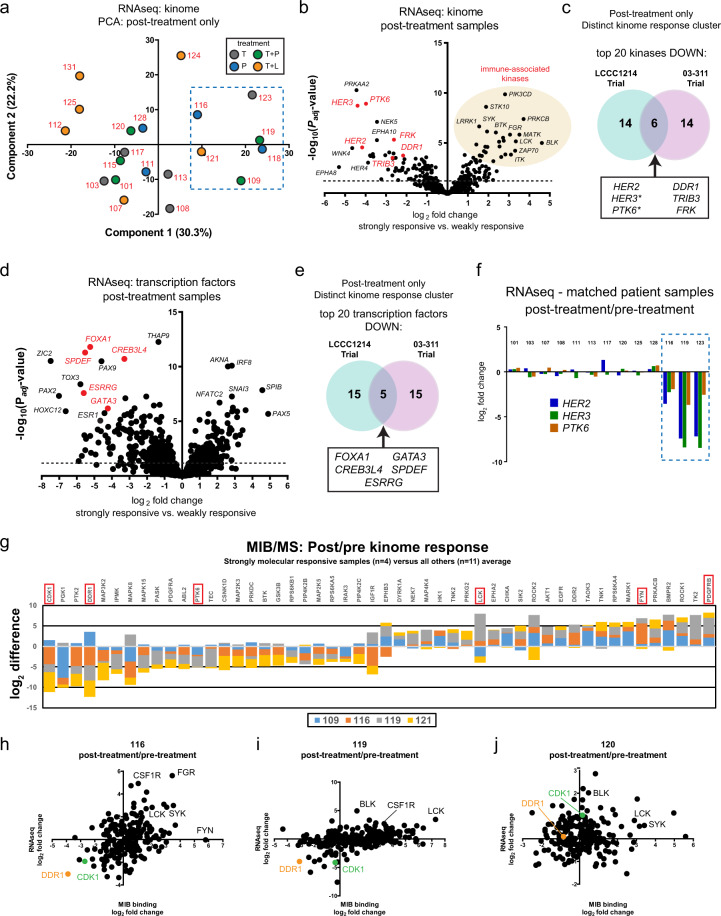
Distinctly responsive post-treatment samples share common kinome and transcriptome features following HER2 targeting. **a** All post-treatment samples with RNAseq data were used to filter for expressed kinases. The normalized kinome expression data was used for principal component analysis (PCA). Patient number is indicated in red for each dot and color indicates LCCC1214 treatment arm. Dashed line indicates previously identified patients 116, 119, and 123 and neighboring samples 109, 118, and 121. **b** RNAseq expression levels of strongly responsive post-treatment samples were compared to the less-responsive post-treatment samples by DESeq2 to identify differentially expressed kinases. The volcano plot indicates the magnitude and the significance of the identified expression differences. Dashed line indicates FDR 5%. **c** Log_2_ normalized gene expression data (GSE76360) of the kinome was analyzed from the 03-311 clinical trial (Clinical trials identifier NCT00148668), in which patient samples were obtained pre-treatment and after 10–14 days of trastuzumab. Post-treatment gene expression data of the kinome was used for unsupervised hierarchical clustering and a segregated cluster of 7 post-treatment patient samples (of 50 total) was identified. Marker selection was performed in Morpheus (Broad Institute) to identify the top 20 kinases significantly down in the strongly responsive post-treatment samples for 03-311 trial data and from DESeq2 analysis of the LCCC1214 trial from (**b**). Venn diagram shows the 6 kinases common to both data sets. Asterisks indicate that more than one gene probe was identified by marker selection for the 03-311 data set (Illumina expression beadchip). **d** DESeq2 analysis of differentially expressed transcription factors in the strongly responsive versus weakly responsive post-treatment LCCC1214 samples. The volcano plot indicates the magnitude and the significance of the identified expression differences. **e** Marker selection was performed in Morpheus (Broad Institute) to identify the top 20 transcription factors with decreased expression in the strongly responsive post-treatment samples for 03-311 trial and compared with the DESeq2 analysis for LCCC1214 from (**a**). The Venn diagram shows the 5 transcription factors common to both clinical data sets. **f** The post-treatment/pre-treatment log_2_ fold change of *HER2*, *HER3*, and *PTK6* expression is shown for paired patient samples. **g** Paired post-treatment and pre-treatment samples from the LCCC1214 study were processed for kinome analysis by MIB/MS. The log_2_ MIB binding changes (post/pre) were plotted as the sum of strongly molecular responsive samples (patients 109, 116, 119, and 121) versus the average difference for weakly-responsive matched pairs and the top 25 differences in both directions are shown. **h**–**j** Log_2_ fold changes for the indicated matched patient samples as determined by MIB binding (*x*-axis) and RNAseq (*y*-axis).

Given the connection between FOXA1, HER2, and HER3 and strong molecular responses in the matched patient samples, we included unmatched clinical trial specimens for further analysis. Initially, principal component analysis (PCA) of the expressed kinome from RNAseq analysis was performed (Fig. 5a). The three distinctly responsive samples (116, 119, and 123) were nearest to three other samples (109, 118, and 121) when their post-treatment kinome expression data was used. Unsupervised hierarchical clustering of all evaluable samples revealed a segregated cluster of the same 6 post-treatment samples (Supplementary Fig. 5a). This response was not exclusive to the kinome, as unsupervised hierarchical clustering based upon the top 2000 differentially expressed genes led to similar segregation (Supplementary Fig. 5b). Importantly, these six post-treatment samples were from two different medical centers, suggesting that the differences were not attributable to technical differences introduced by tissue handling. The distinct group of six post-treatment samples also included a patient specimen from each of the four treatment arms, implying that the transcriptional response was not dependent on a specific HER2 treatment regimen.

Pathway analysis of the distinct post-treatment samples using Enrichr identified a significant enrichment for increased immune response and decreased HER2/HER3 pathway activity associated with the kinase PTK6 and TF FOXA1 (Supplementary Fig. 5c and Supplementary Data 8). Together, the changes in gene expression activity confirmed that this discrete subset of post-treatment patient tumors were dynamically responsive after 7 days of HER2 antibody treatment with evidence of immune infiltration and activation.

To analyze the kinome response, we performed differential expression analysis by DESeq2, comparing the strongly responsive and weakly responsive subsets of post-treatment tumor samples (Fig. 5b and Supplementary Data 9). Kinases with significantly increased expression in the responsive cluster included numerous immune-associated and mesenchymal genes such as *TGFBR2*, *CSF1R*, *LCK*, and *PIK3CD*, consistent with the observed increase in immune gene expression signatures. Kinases with notable changes in significance and magnitude included *HER2*, *HER3*, and *PTK6* (also known as Brk, breast tumor kinase).

We accessed the normalized gene expression data set from a published clinical trial, 03-311 (NCT00148668)^39^. The 03-311 data set included 50 matched pre- and post-treatment sample pairs where HER2+ patients received a single dose of trastuzumab and underwent surgery 7–14 days later. To compare our findings in exclusively post-treatment samples, we initially filtered the data to include only the 50 post-treatment tumor samples and analyzed the expressed kinome (Supplementary Fig. 5d). Unsupervised hierarchical clustering of the kinome expression data from this study identified a subgroup of 7 out of 50 total tumor specimens with a similar profile to the 6 tumors identified in our study. Marker selection analysis on the distinct cluster of 7 tumors versus the remaining 43 was performed and an equivalent comparison was made for our study to highlight any observed similarities (Fig. 5c). Of the top 20 discriminatory kinases with decreased expression, 6 were common to both data sets—*HER2, HER3*, *PTK6*, *DDR1, TRIB3*, and *FRK* (Fig. 5b, red). As the 03-311 expression analysis utilized Illumina beadchip arrays, multiple gene probes were sometimes present. Notably, all probes for *HER3* and *PTK6* were included among the top discriminatory markers for 03-311. We also performed clustering of the log_2_ fold change (post/pre) for the expressed kinases in the 03-311 study. The same 7 patient pairs were close in distance and the response of *HER2* was strongly correlated to *HER3* and *PTK6* (data not shown). Thus, our study and the 03-311 trial both contained a discrete subset of post-treatment patient specimens with markedly stronger transcriptional responses of HER2, HER3, PTK6, and other kinases after 1–2 weeks of HER2 antibody therapy.

We next examined the differentially expressed TFs in the select post-treatment specimens to gain insight into the adaptive response (Fig. 5d). Numerous immune-associated and mesenchymal factors such as *AKNA*, *IRF8*, *IRF4*, and *ZEB2* were significantly increased, consistent with both our global and gene-centric pathway analyses. We compared the expression of TFs in post-treatment samples in our study to the 03-311 trastuzumab study (Fig. 5e). The top 20 discriminatory TFs from the distinctly responsive post-treatment samples in each study revealed five common genes—*FOXA1, GATA3, CREB3L4, SPDEF*, and *ESRRG* (Fig. 5d, red). The ETS family member, SPDEF, exhibits high expression in prostate epithelium and has been shown to promote luminal breast cancer gene expression and proliferation^43,44^. Matched post/pre sample pairs confirmed that the expression of *HER2*, *HER3*, and *PTK6* was reduced (Fig. 5f).

The adaptive kinome response was further interrogated using a chemical proteomic method, MIB/MS, using samples with sufficient tissue for mass spectrometry analysis. Label-free quantification (LFQ) of MIB binding was performed using MaxQuant (MaxLFQ) and the log_2_ fold change for matched post-treatment and pre-treatment pairs was determined (Fig. 5g, Supplementary Fig. 5e, and Supplementary Data 10). Consistent with our observation from gene expression data, a stacked bar plot of the top 20 changes in response to treatment in all samples was enriched for kinases associated with immune cells (e.g., SYK, FGR, FER, BTK). Thus, the kinome of all samples showed evidence of treatment using a standard–of-care HER2 antibody.

To focus on the strongly transcriptionally responsive sample pairs, a stacked bar plot of the 25 kinases with the largest sum differences in MIB binding (decreased and increased) revealed that they were characterized by decreased binding of DDR1, consistent with the RNAseq data, and CDK1, the G_2_/M cyclin-dependent kinase associated with proliferation. Kinases with highest sum increased binding included the mesenchymal marker PDGFRB and immune-associated kinases FYN and LCK in select patients. A scatter plot of log_2_ fold change in MIB binding (*x*-axis) and gene expression (*y*-axis) from two strongly responsive matched patient samples (116, and 119, Figs. 5h and i) and a comparatively weakly responsive matched patient sample (120, Fig. 5j) highlights the consistent increases in immune-associated kinases such as LCK, CSF1R, and SYK. In contrast, the decreases in DDR1 and CDK1 expression and MIB binding were more pronounced in the strongly responsive patient samples. Collectively, analysis of the adaptive kinome response identified an immune response triggered by HER2-targeted therapy and confirmed the loss of key proliferative and breast cancer-associated kinases in the discrete, strongly molecular responsive subset of patients.

### FOXA1-dependent adaptive response gene set applied to patient sample pairs

**Fig. 6:**
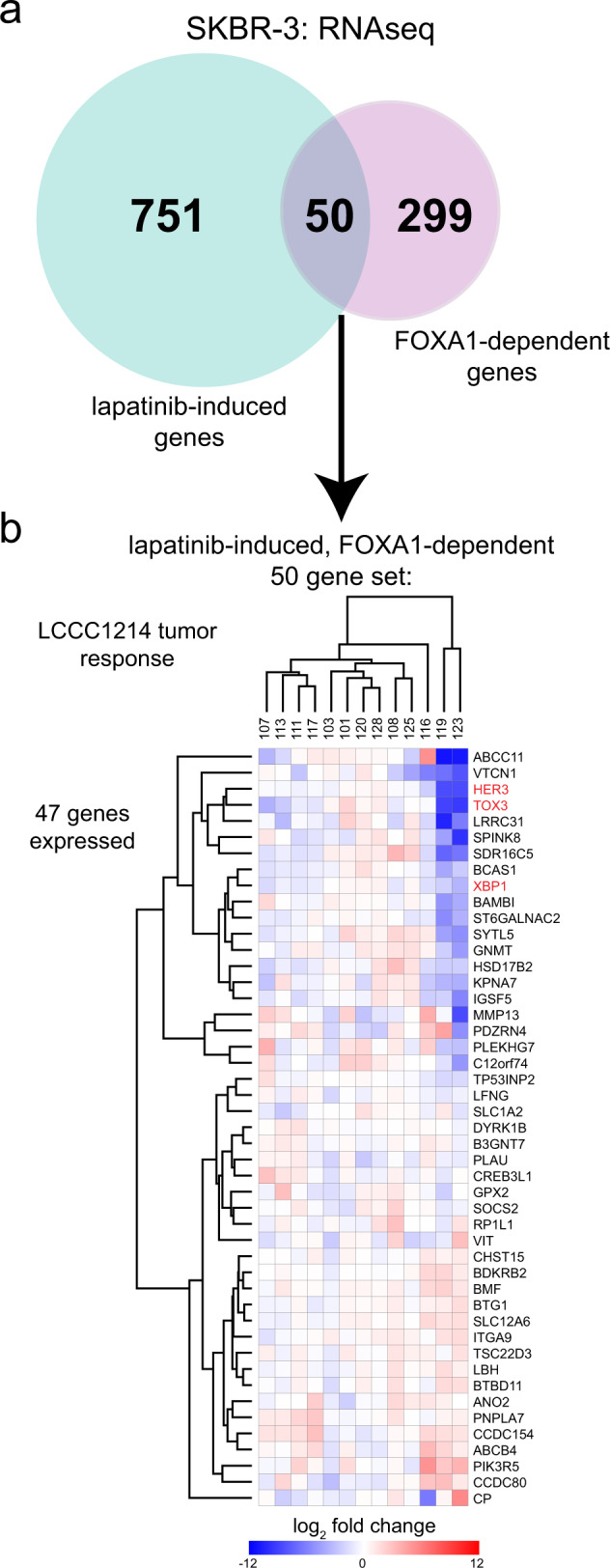
A FOXA1-dependent adaptive response gene set discriminates strongly responsive patient samples. **a** DESeq2 analysis of SKBR-3 cells treated with lapatinib vs. DMSO or with siRNA targeting FOXA1 vs. non-targeting siRNA was performed to identify genes upregulated or downregulated, respectively, with log_2_ fold change of ≥ 1 or ≤ 1, respectively, and FDR cut-off of 5%. The number of genes in each list was compared and the overlap is shown in the Venn diagram to identify FOXA1-dependent genes responsive to HER2 targeting with lapatinib. **b** Log_2_ fold changes in expression for matched patient samples from the gene set identified in (**a**) was used to perform unsupervised hierarchical clustering.

To test the assertion that FOXA1 might regulate genes involved in adaptive response to HER2 inhibition, we used our RNAseq data from SKBR-3 cells treated with lapatinib or treated with FOXA1 siRNA (Fig. 3a). Using DESeq2, we identified genes induced by lapatinib (log_2_ fold change >1, 5% FDR) and genes whose expression was reduced by FOXA1 depletion (log_2_ fold change <1, 5% FDR) and selected the genes present in both sets (Fig. 6a and Supplementary Data 4). When the 50 intersecting genes were used to query Enrichr, significant enrichment was observed for multiple ER and FOXA1 ChIPseq data sets (not shown) which is consistent with the ER–FOXA1 relationship. We next used the 50 genes (“FOXA1-dependent adaptive response genes”), to generate a dendogram by unsupervised hierarchical clustering of matched patient samples (Fig. 6b). Forty-seven of these 50 genes were expressed and HER3 and the transcription factors TOX3 and XBP1 were among the most strongly downregulated genes from this set. In SKBR-3 cells, both TOX3 and XBP1 display the strong induction of proximal SEs following lapatinib treatment that is blocked by JQ1 treatment (Supplementary Fig. 6 and data not shown). We also noted the inclusion of *VTCN1* (V-set domain containing T cell activation inhibitor), a gene responsive to NF kappa B and inflammation and a negative regulator of T cells. Although this gene set was derived from cell type-specific expression changes in HER2+/ER− SKBR-3 cells, the strongly responsive patient samples 116, 119, and 123 could be discriminated. The 20 genes (upper cluster) displaying a trend toward strong reduction post-treatment had a significant association with HER2 from the “ARCHS4 Kinases Coexpression” set after Enrichr query (*P*_adj_ = 0.008050) while the lower cluster (27 genes) did not. The associated genes included *XBP1, SPINK8, BAMBI*, and *VTCN1*. The broad effects on enhancers, gene expression, and phenotype imply that multiple genes may be involved, possibly a TF network, but that FOXA1 reduction elicits a significant response to lapatinib. Taken together, these findings suggest that an ER-independent role is played by FOXA1 in mediating adaptive responses to HER2 inhibition.

## Discussion

HER2 targeting using a variety of available drugs has transformed outcome in this subset of breast cancers. However, resistance remains a problem both in the early and metastatic settings. Moreover, due to the relatively low responsiveness to HER2-targeted drugs alone, the treatment regimens usually include some form of cytotoxic agent. This is the reason that optimizing the effectiveness of all-biologic regimens and identifying the basis for exceptional response is so attractive. Our integrative analysis in HER2+ models implicated FOXA1 as a mediator of gene expression and lineage, irrespective of ER status. While both RNAseq expression analysis and proteomic analysis of the kinome revealed immune responses characteristic of HER2 antibody treatment, we observed a distinct subset of HER2+ patient tumors with comparatively stronger molecular responses. These specimens exhibited dynamic immune pathway alterations and more dramatic reduction in *FOXA1, HER2, HER3* and proliferative kinases such as *CDK1*. Dramatically higher responses in HER2, HER3, and FOXA1 seen in a subset of post-treatment samples was also seen in a subset of patients from an independent clinical trial. We observed that higher HER2 amplicon pathway was associated with early molecular responses. Immune cell activation has been consistently associated with clinical and molecular response to HER2-directed therapy; we also found increases in immune signatures, as well as PD-1 and PD-L1 gene expression 7 days after initiating treatment (Supplementary Fig. 7).

FOXA1 is predominantly considered a pioneer factor for ER from studies in ER+ breast cancer as well as the androgen receptor (AR) in prostate cancer, promoting chromatin accessibility for these TFs^28,34,45^. The expression of FOXA1 correlates with ER expression, with better prognosis, and is amongst the classifier genes in the PAM50 intrinsic subtype classifier^46^. The shared cell lineage of HER2+ breast cancer with other luminal breast cancers prompted deeper investigation into a potential role for FOXA1 in HER2+/ER− breast cancer^47^. FOXA1 was recently identified as an essential gene in luminal breast cancer cell lines^48^. Based on our findings, we propose that FOXA1 is key to HER2+ breast cancer cell identity and adaptive reprogramming. The notion of transcription factor addiction may be true for FOXA1 in HER2+/ER− breast cancer. The recent integration of large data sets to identify dependencies, depMap (https://depmap.org/portal/), further supports that FOXA1 is embedded in breast epithelial lineage, with enriched dependencies for ER+ and HER2+ cell populations. Given that FOXA1 has been shown to function in response to proinflammatory signaling and endocrine resistance in breast cancer cells and in regulatory T cells^49–51^, it will be critical to discriminate FOXA1-dependent, cell type-specific effects. Recruitment of FOXA1 to specific regions, such as the *HER3* intronic enrichment in response to lapatinib observed here (Supplementary Fig. 3) merits further functional studies to establish the requirement of these interactions for HER3 induction.

Recently, Toska et al. demonstrated the cooperative interplay of FOXA1, PBX1, and the histone methyltransferase KMT2D, in the adaptive response of ER+ breast cancer to PI3K inhibition^52^. Epigenetic alterations are a key source of acquired resistance to HER2-directed therapy and potential targetability^53–57^. Trials combining HDAC inhibitors with anti-HER2 drugs have had mixed results but all are small and tolerability has been an issue, although a recent study using entinostat demonstrated some promise^58^.

Wang et al. recently demonstrated that BET bromodomain inhibitors, including JQ1, could interfere with FOXA1-dependent repression and promote prostate cancer invasion^59^. This is consistent with the dramatic effects we observed with either JQ1 treatment or FOXA1 depletion in combination with HER2 targeting (Figs. 1g and 2h). This observation should prompt deeper investigation into both HER2+ and ER+ breast cancer models to elucidate the potential impact of BET bromodomain inhibitors on FOXA1 function. Ongoing and recently completed Phase I studies of BET inhibitors in multiple solid tumors (NCI02259114, NCT03220347, and NCT03035591) suggest that combinations could be employed with HER2-targeting agents. A recent study from Gao et al. demonstrated that demethylation of lysine 270 of FOXA1 by LSD1 was critical for FOXA1 function in prostate cancer models^60^. A small molecule inhibitor of LSD1 (GSK2879552) was effective alone and in combination with AR-targeting enzalutamide in prostate cancer xenograft models. It remains to be determined whether similar effects can be observed using LSD1 inhibition in breast cancer models.

We are aware that limitations of this study include (1) the size of the window-of-opportunity trial, making it not feasible to identify variations in adaptive response mechanisms by drug combination (antibody alone or antibody plus small molecule, for example), and (2) by virtue of the short duration of drug exposure in this trial, it is not possible to associate adaptive changes with clinical endpoints. Nonetheless, this study builds upon existing and emerging evidence of the nature and importance of short-term adaptive responses to kinase inhibition. Our prior work in both triple-negative and HER2+ breast cancer suggest heterogeneity in adaptive reprogramming makes targeting the response challenging; in this study we have found in HER2+ breast tumors that targeting underlying epigenetic mechanisms of reprogramming may be feasible and circumvent variability in expressed adaptations. Like other investigators, we found that immune cell response to anti-HER2-therapy occurs very early; we also found that this short-term response was similar across several anti-HER2 drugs. The other major early and prominent adaptive response is related to FOXA1 and our findings suggest a role for this master TF in adaptive response to HER2-targeted therapy. Thus, ongoing efforts to disrupt FOXA1 as a means to augment treatment of ER+ breast cancer may be useful for HER2+/ER− disease as well.

## Methods

### LCCC1214/TBCRC 036 window-of-opportunity clinical trial

The LCCC1214/TBCRC 036 window trial, “Defining the HER2 Positive (+) Breast Cancer Kinome Response to Trastuzumab, Pertuzumab, Combination Trastuzumab + Pertuzumab, or Combination Trastuzumab + Lapatinib” is registered under the ClinicalTrials.gov identifier NCT01875666 (date of registration, 6/13/2014). GlaxoSmithKline generously provided lapatinib (Tykerb™) and Genentech, Inc. generously provided trastuzumab (Herceptin™) and pertuzumab (Perjeta™) for the study. Eligible women included those with newly diagnosed Stage I–IV HER2+ breast cancer scheduled to undergo definitive surgery (either lumpectomy or mastectomy). Stage I–IIIc patients could not be candidates for a therapeutic neoadjuvant treatment. Histological confirmation of HER2+ status was determined by IHC 3+ or by fluorescence in situ hybridization (FISH), clinical assays on either primary or metastatic tumor. Study subjects provided informed written consent that included details of the non-therapeutic nature of the trial, and the study was approved by the UNC Office of Human Research Ethics and conducted in accordance with the Declaration of Helsinki. Twenty-two patients were enrolled at three institutions (UNC, Dana Farber, MD Anderson) between 10/31/2013 and 12/6/2016, of whom 15 were randomized to a treatment arm (4A, 3B, 4C, 4D). The remaining 7 either declined a required study procedure after enrollment or were found ineligible (e.g., ineligible based on pretreatment laboratory values), some of whom had research biopsies already collected on an institutional tissue banking protocol so could contribute pre-treatment samples for analysis but could not contribute post-treatment samples. No patient had a treatment-related adverse event during the course of study treatment or follow-up. The study was closed for completion of enrollment for this purely correlative trial. For 13 of these patients, matched pre-treatment and post-treatment samples were collected while for 8 additional patients, only a single pre-treatment or post-treatment sample of sufficient quality was available.

Following enrollment, patients were randomly assigned to one of four treatment arms; (A) single dose trastuzumab (8 mg/kg IV); (B) single dose pertuzumab (840 mg); (C) combination trastuzumab (8 mg/kg) + pertuzumab (840 mg) for one dose each; or (D) combination single dose trastuzumab plus oral lapatinib (1000 mg daily) for one week. Study subjects underwent core biopsy of the breast tumor (pre-treatment), received treatment for 7 days prior to the scheduled surgery date. At surgery, a post-treatment tumor specimen was reserved for research. Biopsy and surgical specimens were immediately placed into liquid nitrogen. Flash-frozen pre- and post-treatment samples were processed for RNAseq and for kinome profiling by MIB/MS to evaluate adaptive response to HER2-targeted therapy.

### Next-generation RNA sequencing

RNA was extracted from frozen patient specimens, cell lines, or frozen PDX tumors using Qiagen RNeasy Plus Kit with optional DNase I treatment. Libraries were prepared using the KAPA Stranded mRNAseq kit according to the manufacturer’s protocol with 12 cycles of PCR amplification. Samples were indexed with Illumina TruSeq adaptors. Samples were run on an Illumina NextSeq 500 using High Output Kits to produce single end 75 bp or paired-end 50 bp reads.

### RNAseq analysis and PAM50 intrinsic subtyping

FastQC-passed reads were aligned to the human reference genome (hg38) using STAR 2.4.2a^61^ and reads were translated to transcriptome coordinates using Salmon 0.60^62^. Isoform data were collated to single gene IDs using the R package biomaRt^63^, and abundance estimates were upper quartile normalized using R. Gene values were filtered to include only those with 10 or more reads in at least one sample and values (x + 1) were log_2_ transformed and used for unsupervised hierarchical clustering (complete linkage) in Morpheus (https://software.broadinstitute.org/morpheus/). DESeq2 was used for differential expression analysis using raw counts and an FDR cut-off of 5% was used to filter genes used for Enrichr analysis^64,65^. LCCC1214 samples went through standard PAM50 algorithm, samples were classified into 5 different breast cancer subtypes based on the 50 intrinsic gene scores and classification^66^.

### RNAseq pathway signature analysis

In order to implement each signature, the methods detailed in the original studies were followed as closely as possible^67^. The gene expression data set was log_2_ transformed, median centered, and filtered to exclude genes with missing value in more than 20% of the samples. The mean expression value was then calculated using all genes within a given signature and the resulting signature scores are reported in the Supplementary Data 5. Statistical analysis was performed using SAM (*q* < 0.05) to identify significant differences in pathway activity between strong and weak responsive post-treatment samples as well as pre-treatment samples for the matched pairs. Unsupervised hierarchical clustering of pathway signatures (complete linkage) was performed using Gene Cluster 3.0 and Java Treeview was used to visualize the resultant heat maps.

### MIB chromatography, LC-MS/MS, and analysis

Flash-frozen tumor samples were crushed by mortar and pestle in ice-cold MIB lysis buffer and MIB chromatography performed as previously described. Extracts were sonicated 3 × 10 s, clarified by centrifugation, and syringe-filtered (0.22 μm) prior to Bradford assay quantitation of concentration. Equal amounts of total protein (0.3 mg) were gravity-flowed over multiplexed inhibitor bead (MIB) columns in high salt MIB lysis (1 M NaCl). The MIB columns consisted of 175 μl mixture of six Type I kinase inhibitors (CTx0294885, VI-16832, PP58, Purvalanol B, UNC-21474, and UNC-8088A) custom-synthesized with hydrocarbon linkers and covalently linked to ECH-Sepharose (or EAH-Sepharose for Purvalanol B) beads as previously described^12,19^. Columns were washed with 5 mL of high salt (1 M NaCl), 5 mL of low salt (150 mM NaCl) MIB lysis buffer, and 0.5 mL low-salt lysis buffer with 0.1%SDS. Bound protein was eluted twice with 0.5% SDS, 1% beta-mercaptoethanol, 100 mM Tris-HCl, pH 6.8 for 15 min at 100 °C. Eluate was treated with DTT (5 mM) for 25 min at 60 °C and 20 mM iodoacetamide for 30 min in the dark. Following spin concentration using Amicon Ultra-4 (10k cut-off) to ~100 μL, samples were precipitated by methanol/chloroform, dried in a speedvac and resuspended in 50 mM HEPES (pH 8.0). Tryptic digests were performed overnight at 37 °C, extracted four times with 1 mL ethyl acetate to remove detergent, dried in a speed-vac, and peptides further cleaned using C-18 spin columns according to the manufacturer’s protocol (Pierce). Peptides were resuspended in 2% ACN and 0.1% formic acid. 40% of the final peptide suspension was injected onto a Thermo Easy-Spray 75 μm × 25 cm C-18 column and separated on a 120 min gradient (5–40% ACN) using an Easy nLC-1200. The Thermo Q Exactive HF mass spectrometry ESI parameters were as follows: 3e6 AGC MS1, 80 ms MS1 max inject time, 1e5 AGC MS2, 100 ms MS2 max inject time, 20 loop count, 1.8 *m/z* isolation window, 45 s dynamic exclusion. Raw files were processed for label-free quantification by MaxQuant LFQ using the Uniprot/Swiss-Prot human database and default parameters were used with the following exceptions—only unique peptides were used, carbidomethyl (C) fixed modification, and phospho (STY) dynamic modifications, and matching between runs was utilized. In Perseus software (Max Planck Institute), LFQ intensities were log_2_-transformed and missing values were imputed by column using default parameters.

### Cell lines, authentication, and in vitro treatment

SKBR-3 and BT474.m1 HER2+ breast cancer lines were grown in RPMI 1640 supplemented with 10% fetal bovine serum and 1% penicillin/streptomycin. These lines obtained from UNC Lineberger Tissue Culture Facility or collaborators, cultured no more than 6 months, and routinely checked for mycoplasma contamination by DAPI staining. Cells were treated for 24 h with lapatinib (300 nmol/L final concentration) or JQ1 (300 nmol/L final concentration) purchased from Selleck Chemicals and dissolved in DMSO.

### Chromatin immunoprecipitation, library preparation, and analysis

Chromatin immunoprecipitation (ChIP) experiments were as described previously^20^. Briefly, 1 × 10^7^ cells per IP were crosslinked for 10 min at room temperature in 1% formaldehyde and neutralized with a final concentration of 125 mM glycine. Nuclear extracts were sonicated 15 cycles (30 s pulse, 30 s cooling) using a Bioruptor Pico (Diagenode). Samples were tumbled overnight at 4 °C with 10 μg antibody (control rabbit IgG (ThermoFisher, Cat. 02-6102), anti-BRD4 (Bethyl Laboratories, Cat. A301-985A100), anti-MED1 (Bethyl Laboratories, Cat. A300-793A100), anti-H3K27ac (Active Motif, Cat. 39133), or anti-FOXA1 (Abcam, Cat. ab5089) conjugated to protein A Dynabeads (ThermoFisher, Cat. 10006D)). After washing and RNaseA/proteinase K treatment, DNA was purified using a Qiagen MinElute PCR purification kit. ChIPseq libraries were prepared using the KAPA HyperPrep Kit according to the manufacturer’s protocol using an equal amount of input DNA. Samples were indexed with Illumina TruSeq adaptors and dual size selection was performed following 16–18 cycles of PCR amplification. Samples were run as equimolar 12-plexes on an Illumina NextSeq 500 using a 75 cycle, high output kit to produce single-end 75 bp reads. Analysis was performed essentially as described by Zawistowski et al., except that peaks within 20 kb were stitched and peaks defined by the following criteria: ±5 kb of any transcription start site (TSS) = promoter, −5 to −200 kb of any TSS = 5′ enhancer, if overlapped with gene boundary = genebody_exon or genebody_intron, within 0 to +200 kb from the 3′ most exon and not classified as any other = 3′ enhancer. All other peaks were defined as “other” or “orphan.” Genes defined as “proximal” for comparison to expression data were defined as those genes whose TSS was within 200 kb 5′ of the 5′ edge or 200 kb 3′ of the 3′ edge of an induced ChIPseq peak.

### siRNA transfection, cell proliferation, and western blotting

Dharmacon (now Horizon Discovery) siGENOME SMARTPool siRNAs targeting MYC (Catalog ID:M-003282-07-0005), FOXA1 (Catalog ID:M-010319-01-0005), FOXO3 (Catalog ID:M-003007-02-0005), FOXO1 (Catalog ID:M-003006-03-0005), or Non-Targeting Control Pool #2 (Catalog ID:D-001206-14-05) were reverse transfected at a final concentration of 25 nM with RNAiMAX (Life Technologies). siRNAs were mixed with 1/1000 final volume RNAiMAX in serum- and antibiotic-free IMDM and incubated at room temperature for 20–30 min before plating trypsinized cells in RPMI 1640 media with 10% FBS and antibiotics. For drug treatment, cells were incubated in siRNA for 24 h before replacement of media with DMSO or drug-containing media. Cells were plated (SKBR-3: 4,000 cells/well and BT474.m1: 6000 cells/well) in 96-well plates and drug-containing media was replenished every 24 h. Live cells were stained with Hoechst in PBS for 20 min at 37 degrees C and imaged/counted with a Thermo Cellomics ArrayScan VTI at 25 frames per well. Equal amounts of extracted protein (determined by Bradford assay) were separated by SDS-PAGE, transferred to nitrocellulose and probed with anti-BRD4 (Bethyl Laboratories, Cat. A301-985A100, 1:1000), anti-ERK2 (Santa Cruz Cat. sc-1647, 1:2000), anti-FOXA1 (Abcam, Cat. ab5089, 1:1000) or the following antibodies (all with Cell Signaling Cat. numbers and used at 1:1000): anti-FOXO1 (2880), anti-FOXO3 (2497), anti-phospho-HER2/ErbB2 (Tyr1221/1222) (2243), anti-HER2 (2242), anti-HER3 (4754), anti-phospho-AKT (Thr308) (4056), anti-phospho-AKT (Ser473) (4060), anti-phospho-ERK1/2 (4370). All western blots shown derive from the same experiment, were processed in parallel, and are representative of at least two experiments. Uncropped images are included in the Supplementary Material.

### Reporting summary

Further information on research design is available in the Nature Research Reporting Summary linked to this article.

## Supplementary information

Supplementary Information

Supplementary Data 1

Supplementary Data 2

Supplementary Data 3

Supplementary Data 4

Supplementary Data 5

Supplementary Data 6

Supplementary Data 7

Supplementary Data 8

Supplementary Data 9

Supplementary Data 10

Reporting Summary

## Data Availability

The data generated and analyzed during this study are described in the following data record: 10.6084/m9.figshare.14376746^68^. The mass spectrometry proteomics data have been deposited to the ProteomeXchange Consortium via the PRIDE partner repository with the data set identifier https://identifiers.org/pride.project:PXD021865^69^. Normalized patient RNAseq data (https://identifiers.org/geo:GSE161743), cell line RNAseq (https://identifiers.org/geo:GSE160001 and https://identifiers.org/geo:GSE160001), and cell line ChIPseq (https://identifiers.org/geo:GSE160667) are all part of the SuperSeries https://identifiers.org/geo:GSE160670^70^ available through the Gene Expression Omnibus. Processed and normalized data are provided as supplemental materials and also reusable format with the data record^68^. Accompanying Supplementary Information and Supplementary Data files contain relevant data used to produce the included figures and are available with this article. A detailed list of which data files underlie which figures and tables in the related article is included in the file ‘Angus_et_al_2021_underlying_data_files_list.xlsx’, which is shared with the data record^68^.

## Code availability

Python code generated in the laboratory for ChIPseq analysis is available at GitHub https://github.com/darshansinghunc/chippeakanalysis.
