# Supplementary material for: FOXA1 and adaptive response determinants to HER2 targeted therapy in TBCRC 036
Source: NPJ Breast Cancer. 2021 May 12;7:51. doi: 10.1038/s41523-021-00258-0 (PMC8115531; doi:10.1038/s41523-021-00258-0)
Supplement: Supplementary file 1 — Supplementary Information [file 41523_2021_258_MOESM1_ESM.pdf]

SUPPLEMENTARY INFORMATION – CONTENTS – Angus et al.

Supplementary Figures 1-7, legends below Figures.

Supplementary Data 1-8 Legends

Uncropped western blot images related to Fig. 1f.

Uncropped western blot images related to Fig. 3b.

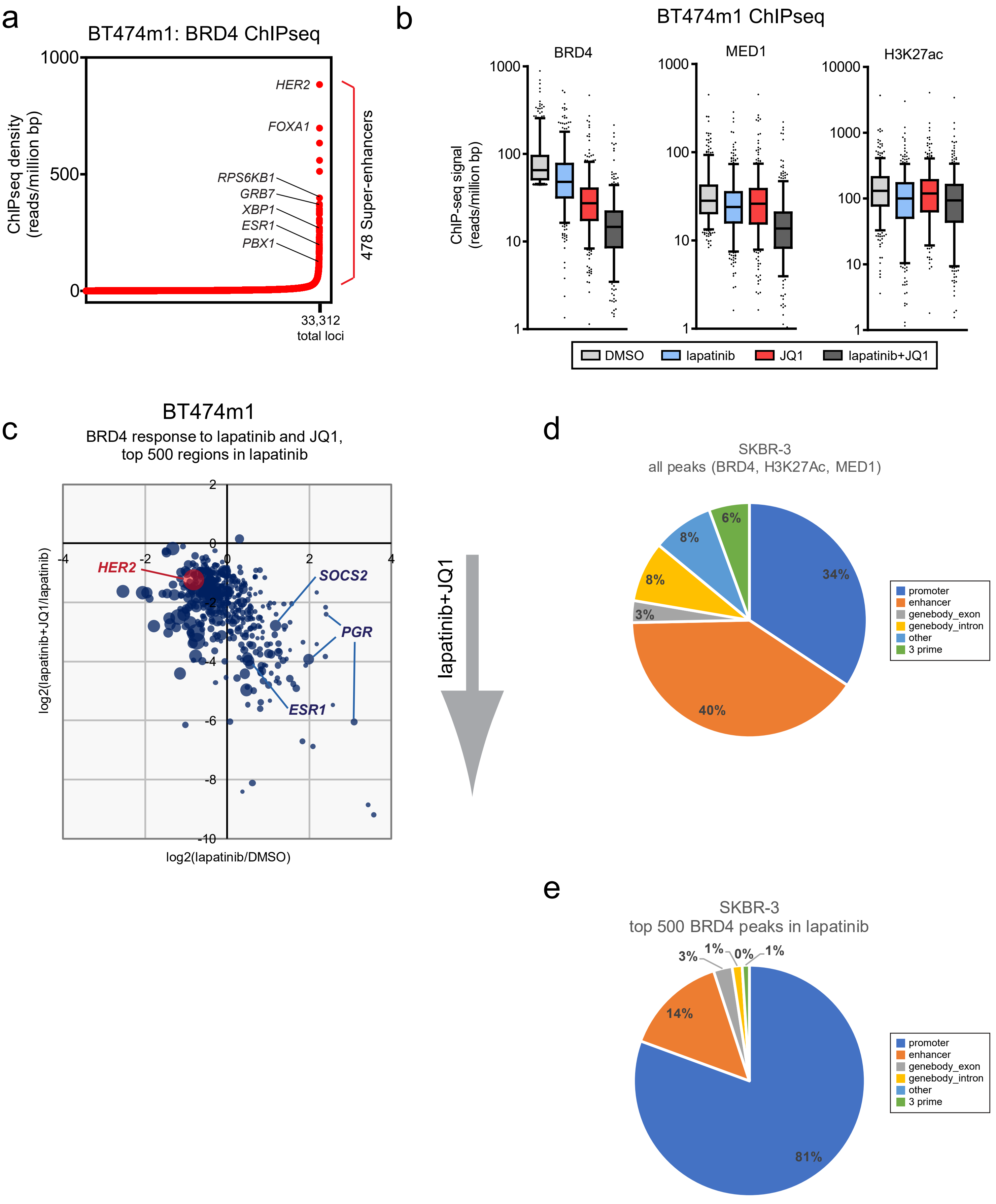

Supplementary Figure 1

Supplementary Figure 1. Lapatinib and JQ1 cooperatively disrupt SEs in BT474.m1 cells (A) ChIPseq analysis of the ERBB2+ breast cancer cell line, BT474.m1, was performed to identify BRD4 binding sites. Super-enhancers (478 regions) were identified as regions above the inflection point of increasing BRD4 ChIPseq density. (B) BRD4 and MED1 chromatin association is significantly reduced by the combination of lapatinib and JQ1, but H3K27Ac is unaffected. Box plots: median, upper/lower quartile, and 5-95 percentile. (C) The combination of lapatinib and JQ1 cooperatively reduce BRD4 binding to ERBB2. Lapatinib-induced binding of BRD4 to ESR1 (ER), SOCS2, and PGR are reduced by the addition of JQ1. The top 500 regions of highest ChIPseq density in response to lapatinib are shown for BRD4. The log2 fold change in density in response to lapatinib vs. DMSO is plotted on the x-axis. The log2 fold change of lapatatinib+JQ1 vs. lapatinib alone is plotted on the y-axis. Dot size is relative to ChIP density in lapatinib. (D) Peak classification distribution for BRD4, MED1, and H3K27Ac ChIPseq results from SKBR-3 cells (DMSO control). (E) Top 500 peak classification distribution for BRD4 ChIPseq results from SKBR-3 cells in response to 24 h 300nm lapatinib treatment.

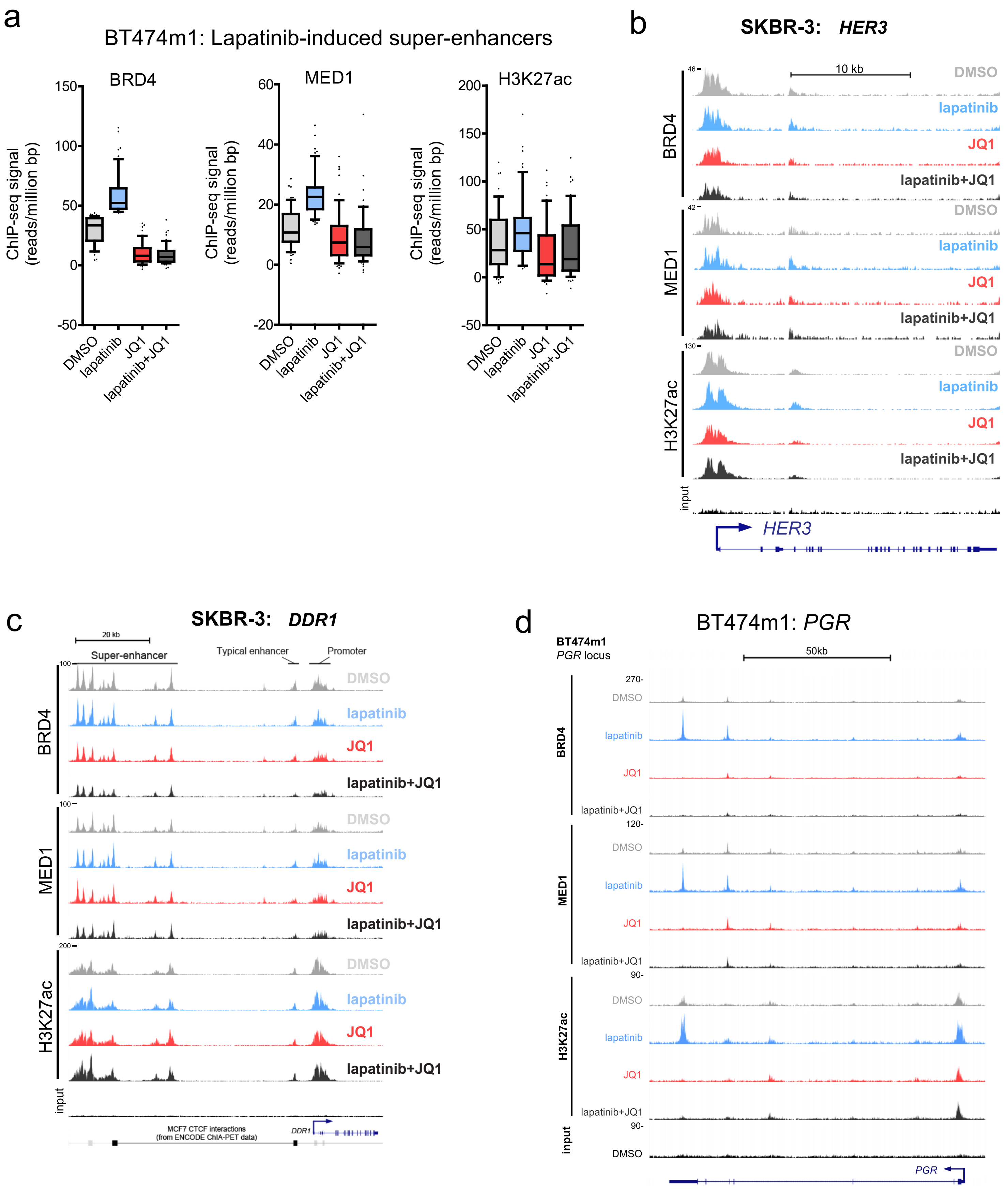

Supplementary Figure 2

Supplementary Figure 2. JQ1 addition opposes lapatinib-induced chromatin binding at enhancers in BT474.m1 cells but de novo SEs are not formed at transcriptionally responsive kinase genes in SKBR-3 cells. (A) Lapatinib-induced SEs, as determined by BRD4 ChIPseq density in BT474.m1 cells. Lapatinib in combination with JQ1 suppresses BRD4 binding in all regions, MED1 in most regions, but does not affect H3K27Ac. Box plots: median, upper/lower quartile, and 5-95 percentile. (B) and (C) The ERBB3 and DDR1 loci, displaying BRD4, MED1, and H3K27Ac ChIPseq density from SKBR-3 cells in response to lapatinib, JQ1, or the combination. (D) The PGR (progesterone receptor, PR) locus, displaying BRD4, MED1, and H3K27Ac ChIPseq density from BT474.m1 cells in response to lapatinib, JQ1, or the combination.

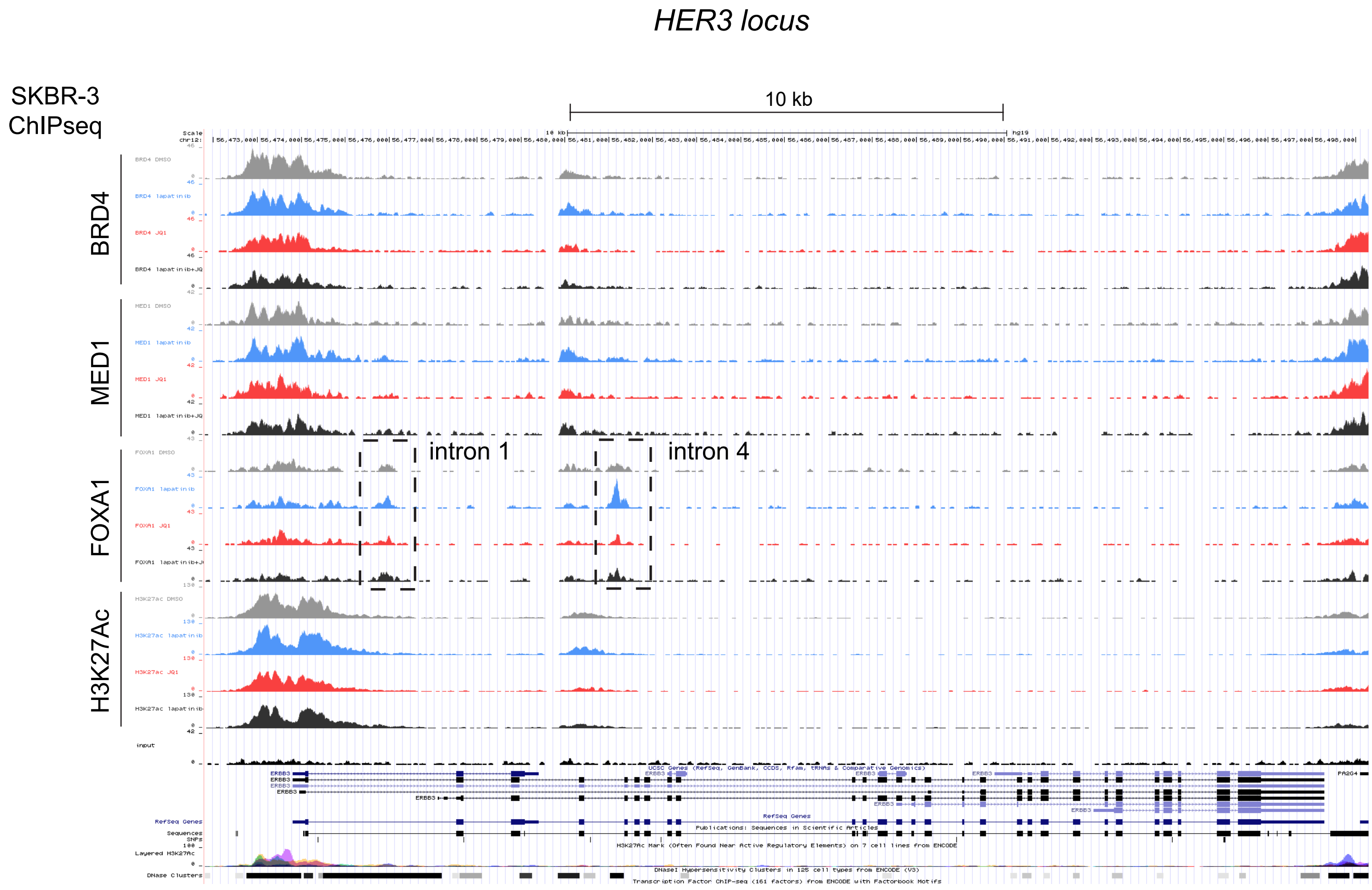

Supplementary Figure 3. Lapatinib induces FOXA1 binding to intron 1 and intron 4 of the ERBB3 locus that is disrupted by JQ1. The XBP1 locus, displaying BRD4, MED1, FOXA1 and H3K27Ac ChIPseq density from SKBR-3 cells in response to lapatinib, JQ1, or the combination. Dotted lines indicate regions of increased FOXA1 binding in intron 1 and intron 4 of ERBB3 in response to lapatinib.

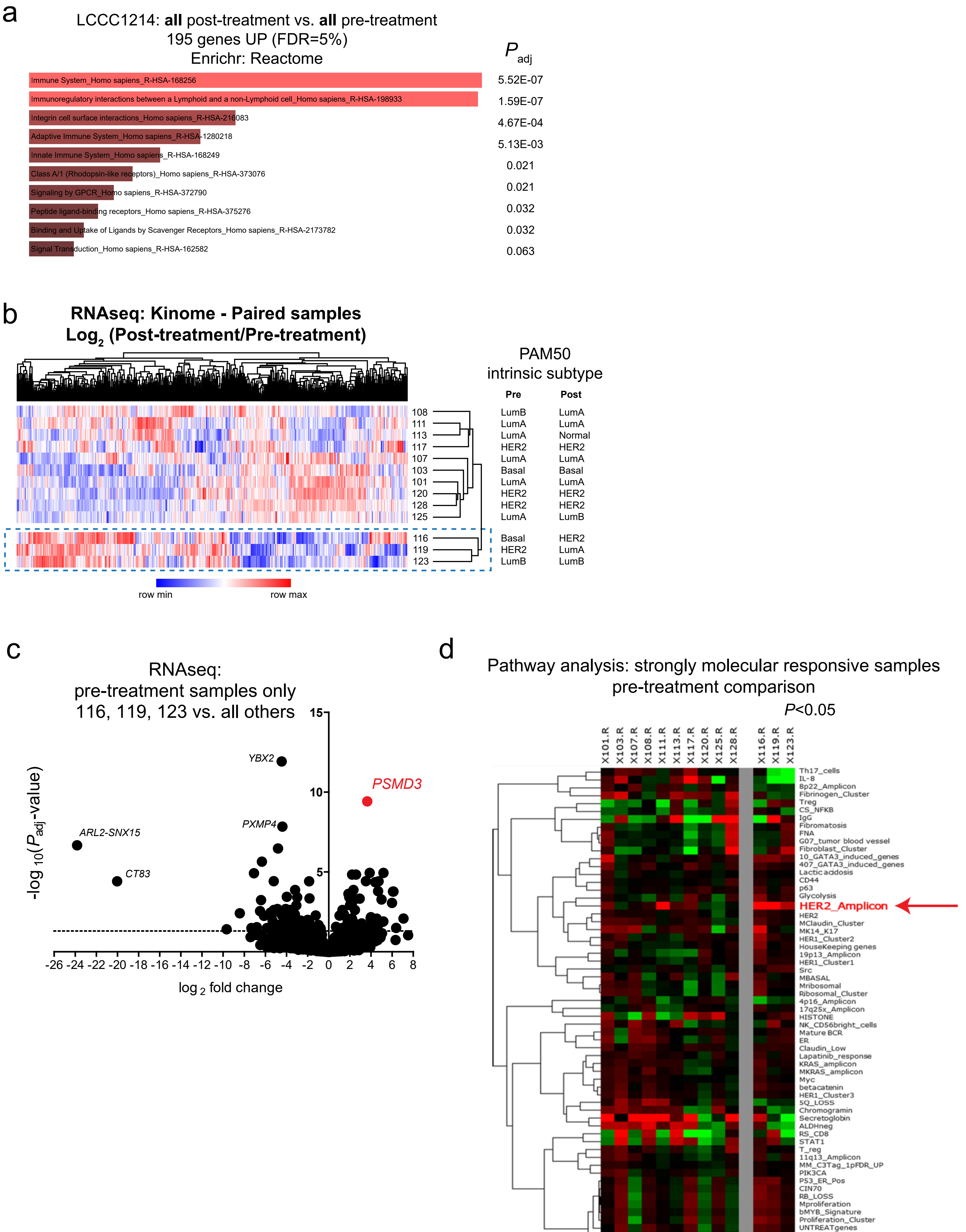

Supplementary Figure 4

Supplementary Figure 4. Evidence for immune infiltration in response to ERBB2 antibody, PAM50 subtypes for matched patient samples, and strongly responsive patient samples have higher ERBB2 amplicon pathway signature at baseline. (A) DESeq2 analysis of all post-treatment versus all pre-treatment samples was performed and all genes with significantly increased expression in the post-treatment samples (5% FDR cutoff) were used to query Enrichr and the results of the Reactome database are shown. (B) Log2 post-treatment/pre-treatment matched-patient ratios of RNAseq normalized reads for kinase genes were used to perform unsupervised hierarchical clustering. A cluster of strongly responsive post-/pre-treatment patient samples is indicated by blue dashed line. PAM50 intrinsic subtype was determined for each pre-treatment and post-treatment sample and the classifications are indicated. (C) DESeq2 was used to identify differentially expressed genes comparing the pre-treatment samples for patients 116, 119, and 123 (strongly responsive after treatment) to all other pre-treatment samples. The volcano plot indicates the magnitude and the significance of the identified expression differences. PSMD3, a gene found on the ERBB2 amplicon, is shown in red. (D) Pathway analysis was performed comparing the strongly responsive pre-treatment samples (116, 119, 123) to all other pre-treatment samples. Unsupervised hierarchical clustering of the pathway scores was performed for the significantly different pathways (unpaired t-test, $P<0.05$).

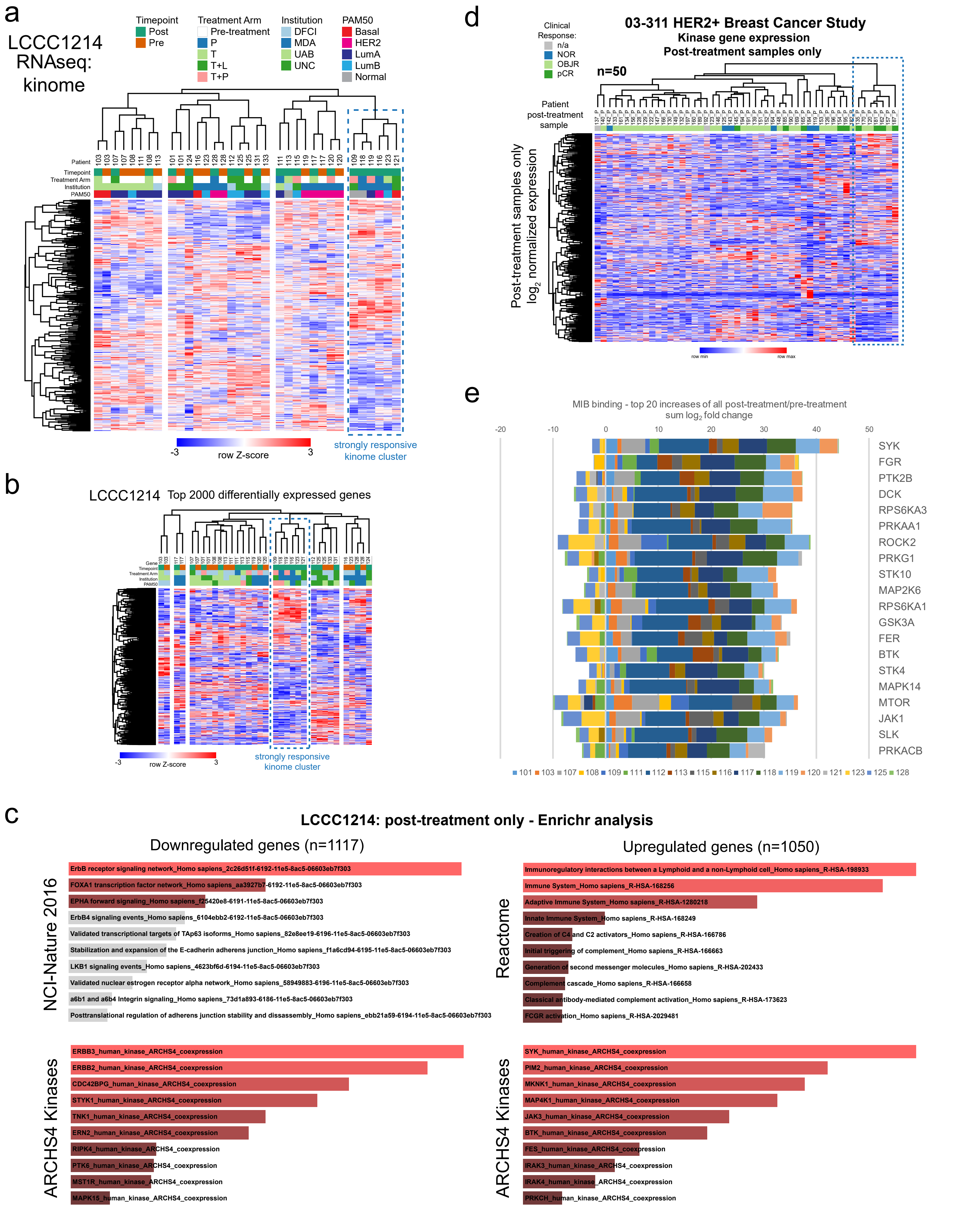

Supplementary Figure 5

Supplementary Figure 5. Shared kinome and transcriptome features in strongly responsive samples links FOXA1, ERBB3, ERBB2, and PTK6 reduction with evidence of immune response. (A) All accessible and evaluable patient pre-treatment and post-treatment tumor samples were processed for RNAseq. Log2 normalized reads of the expressed kinome were quantile normalized, mean-centered, and unsupervised hierarchical clustering was performed. PAM50 intrinsic breast cancer subtype classification, clinical time point, treatment arm, and clinical site are indicated. A distinct cluster of exclusively post-treatment samples is indicated by blue dashed line. (B) Unsupervised hierarchical clustering of normalized reads of the top 2000 differentially expressed genes from LCCC1214 patient samples, log2 transformed, quantile normalized, and mean-centered. (C) DESeq2 analysis was performed comparing the strongly responsive post-treatment samples (n=6) vs. all other post-treatment samples (n=14). Upregulated and downregulated genes (5% FDR cutoff) with log2 fold change cutoff of 2 or -2 were used to query Enrichr and the indicated pathways are shown with combined score ranking. (D) Log2 normalized gene expression data (GSE76360) of the kinome was analyzed from the 03-311 clinical trial (Clinical trials identifier NCT00148668), in which patient samples were obtained pre-treatment and after 10-14 days of trastuzumab. Post-treatment gene expression data of the kinome was used for unsupervised hierarchical clustering and a segregated cluster of 7 post-treatment patient samples (of 50 total) was identified (dashed line). (E) Paired post-treatment and pre-treatment samples from the LCCC1214 study were processed for kinome analysis by MIB/MS. The top 20 increases in log2 MIB binding (post/pre) are shown as a stacked bar plot.

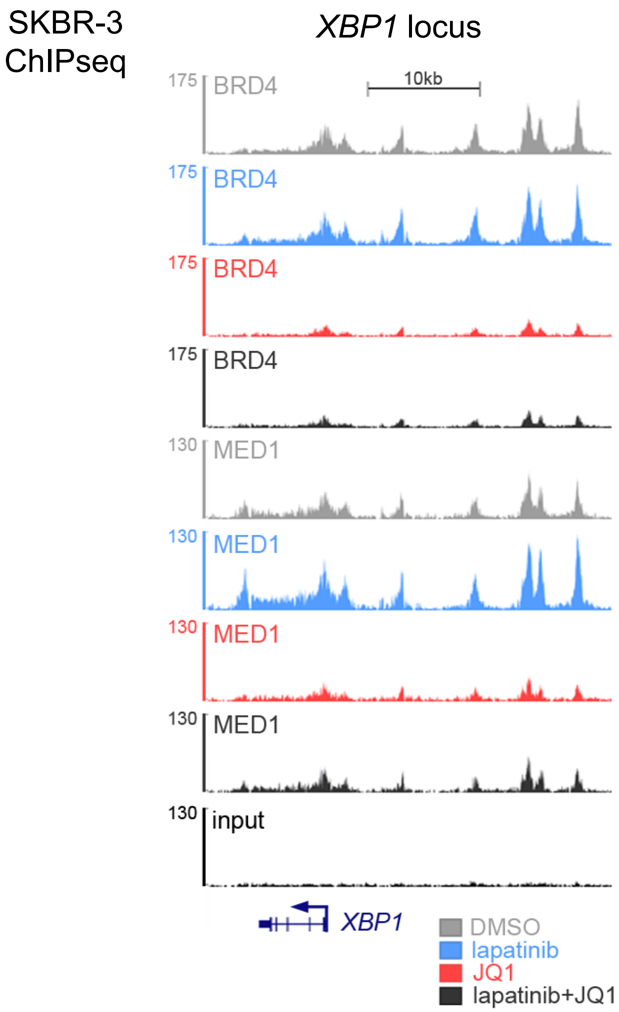

Supplementary Figure 6

Supplementary Figure 6. Lapatinib-induced super enhancer formation at the XBP1 locus. The XBP1 locus, displaying BRD4, MED1, and H3K27Ac ChIPseq density from SKBR-3 cells in response to lapatinib, JQ1, or the combination.

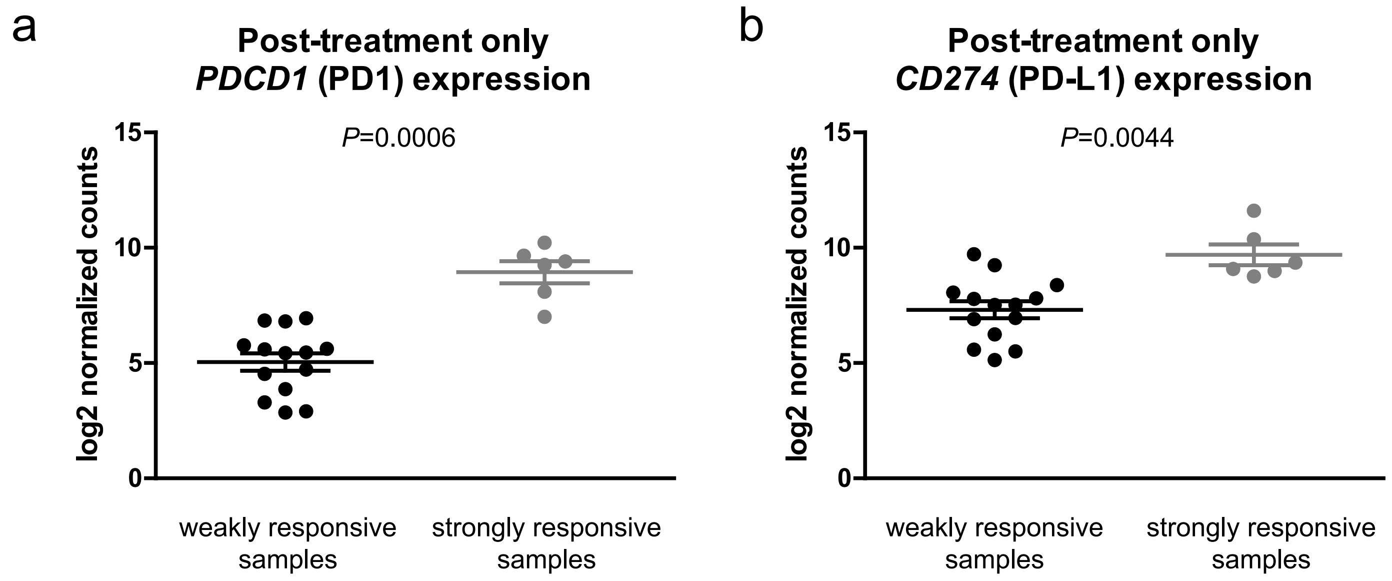

Supplementary Figure 7. Strongly responsive samples have increased PD-1 and PD-L1 expression. Comparison of (A) PDCD1 (also known as PD-1) gene expression and (B) CD274 (also known as PD-L1) in the strongly molecular responsive vs. weakly molecular responsive post-treatment LCCC1214 clinical samples (Mann-Whitney test P-values are shown).

SUPPLEMENTARY DATA LEGENDS

Supplementary Data 1. ChIPseq peak classifications and normalized density (read counts) for SKBR-3 cells. Peak: genome coordinates. Peaksize: in base pairs. Classification: promoter, genebody_exon, genebody_intron, enhancer, 3 prime, other (see Methods for details on classification). Classification gene: annotated gene(s) proximal to peak. Header: Cell line_ChIP antibody_Treatment_Duration. Displayed in Figure 1b-g, Figure 2a-e, Supplementary Figure 1d and 1e, and Supplementary Figure 2b-c, Supplementary Figure 3, and Supplementary Figure 6.

Supplementary Data 2. ChIPseq peak classifications and normalized density (read counts) for BT474.m1 cells. Peak: genome coordinates. Peaksize: in base pairs. Classification: promoter, genebody_exon, genebody_intron, enhancer, 3 prime, other (see Methods for details on classification). Classification gene: annotated gene(s) proximal to peak. Header: Cell line_ChIP antibody_Treatment_Duration. Displayed in Supplementary Figure 1 and Supplementary Figure 2a and 2d.

Supplementary Data 3. ChIPseq peak classifications and normalized density (read counts) for SKBR-3 cells. Peak: genome coordinates. Peaksize: in base pairs. Classification: promoter, genebody_exon, genebody_intron, enhancer, 3 prime, other (see Methods for details on classification). Classification gene: annotated gene(s) proximal to peak. Header: siRNA_ChIP antibody_Treatment_Duration. NT, non-targeting siRNA pool. Displayed in Figure 1h.

Supplementary Data 4. DESeq2 results of SKBR-3 cells treated with FOXA1 siRNA and response to lapatinib. DESeq2 results: comparison (“lapatinib/DMSO”), baseMean, log2FoldChange, lfcSE (standard error), stat (Wald statistic), pvalue (Wald test p-vlaue), padj (BH adjusted p-value). Displayed/used to produce Figure 3a.

Supplementary Data 5. Normalized RNAseq counts from LCCC1214 (TBCRC 036) trial. “Sample Key” tab indicates gene, patient ID as 1xx, timepoint, treatment site (institution), treatment arm, and specific treatment. “norm-counts” contains normalized counts, patient ID, weak vs. strong kinome responseas determined by unsupervised clustering, kinase genes (with kinase family indicated), and if the gene is classified as a transcription factor (TF). Displayed/used to produce Figure 4b-d, Figure 5a-f, Supplementary Figure 4, 5, 7.

Supplementary Data 6. DESeq2 results of all post-treatment versus all pre-treatment patient samples for LCCC1214 (TBCRC 036) trial. baseMean, log2FoldChange, lfcSE (standard error), stat (Wald statistic), pvalue (Wald test p-vlaue), padj (BH adjusted p-value). Displayed in Supplementary Figure 4a.

Supplementary Data 7. DESeq2 results of strong vs. weak molecular response pre-treatment patient samples for LCCC1214 (TBCRC 036) trial . baseMean, log2FoldChange, lfcSE (standard error), stat (Wald statistic), pvalue (Wald test p-vlaue), padj (BH adjusted p-value). Displayed in Supplementary Figure 4c.

Supplementary Data 8. Pathway signature scores from RNAseq data from LCCC1214 (TBCRC 036) trial. Pathway signature scores for patient samples (“1xx”), pre-treatment (“R”) or post-treatment (“O”). Displayed/used to produce Figure 4f and Supplementary Figure 4d.

Supplementary Data 9. DESeq2 results of strongly responsive vs. weakly molecular responsive post-treatment samples from LCCC1214 (TBCRC 036). baseMean, log2FoldChange, lfcSE (standard error), stat (Wald statistic), pvalue (Wald test p-vlaue), padj (BH adjusted p-value). Displayed/used to produce Figure 5b-e and Supplementary Figure 5.

Supplementary Data 10. Kinome profiling (MIB/MS) log2 difference in label-free quantification (LFQ) intensities for post-treatment vs. pre-treatment matched patient samples. Kinase, patient IDs, and log2 LFQ difference from LCCC1214 (TBCRC 036). Displayed/used to produce Figure 5g-j.

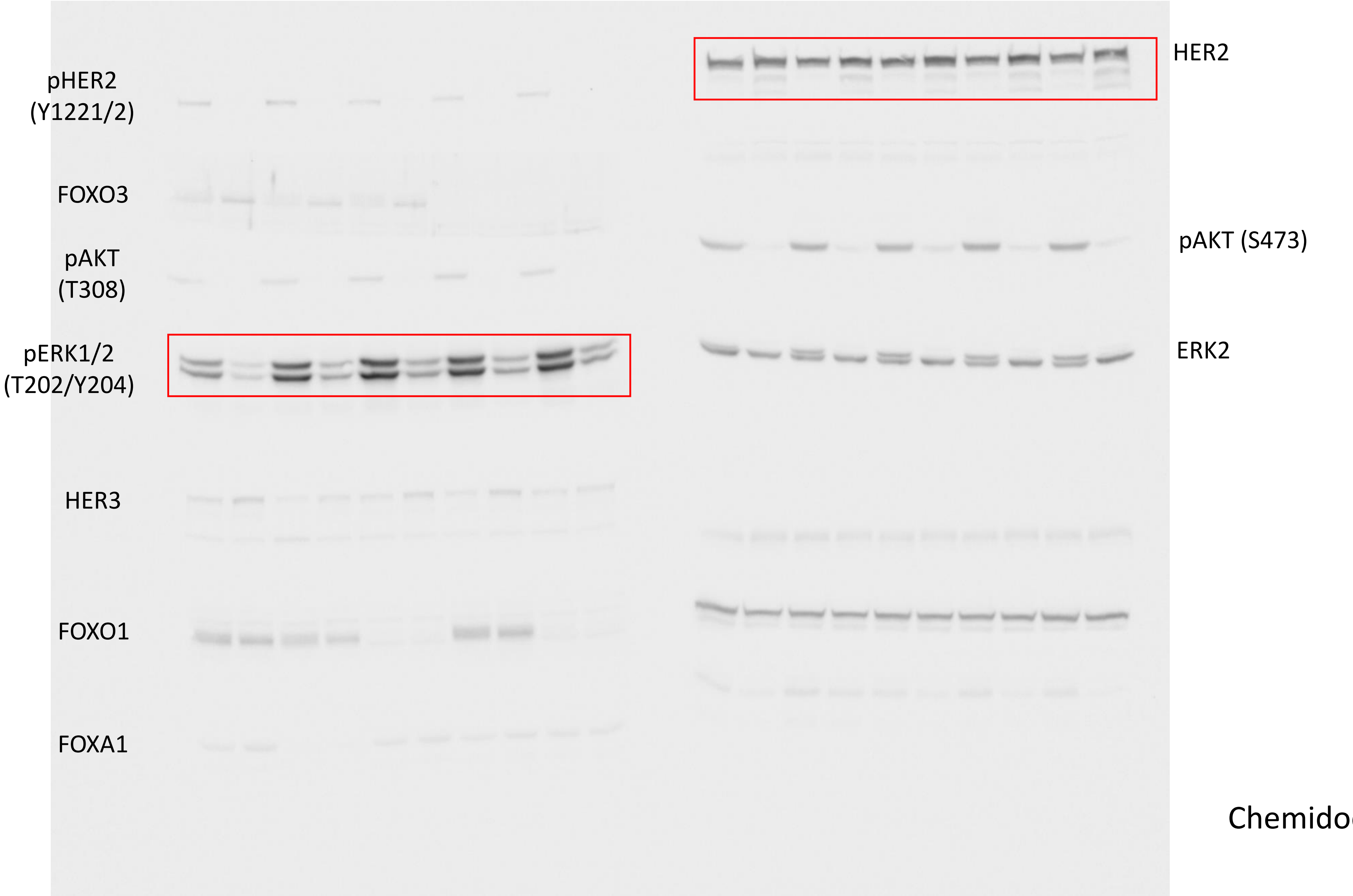

Chemidoc Scans – related to Fig. 3b

Exposure 1

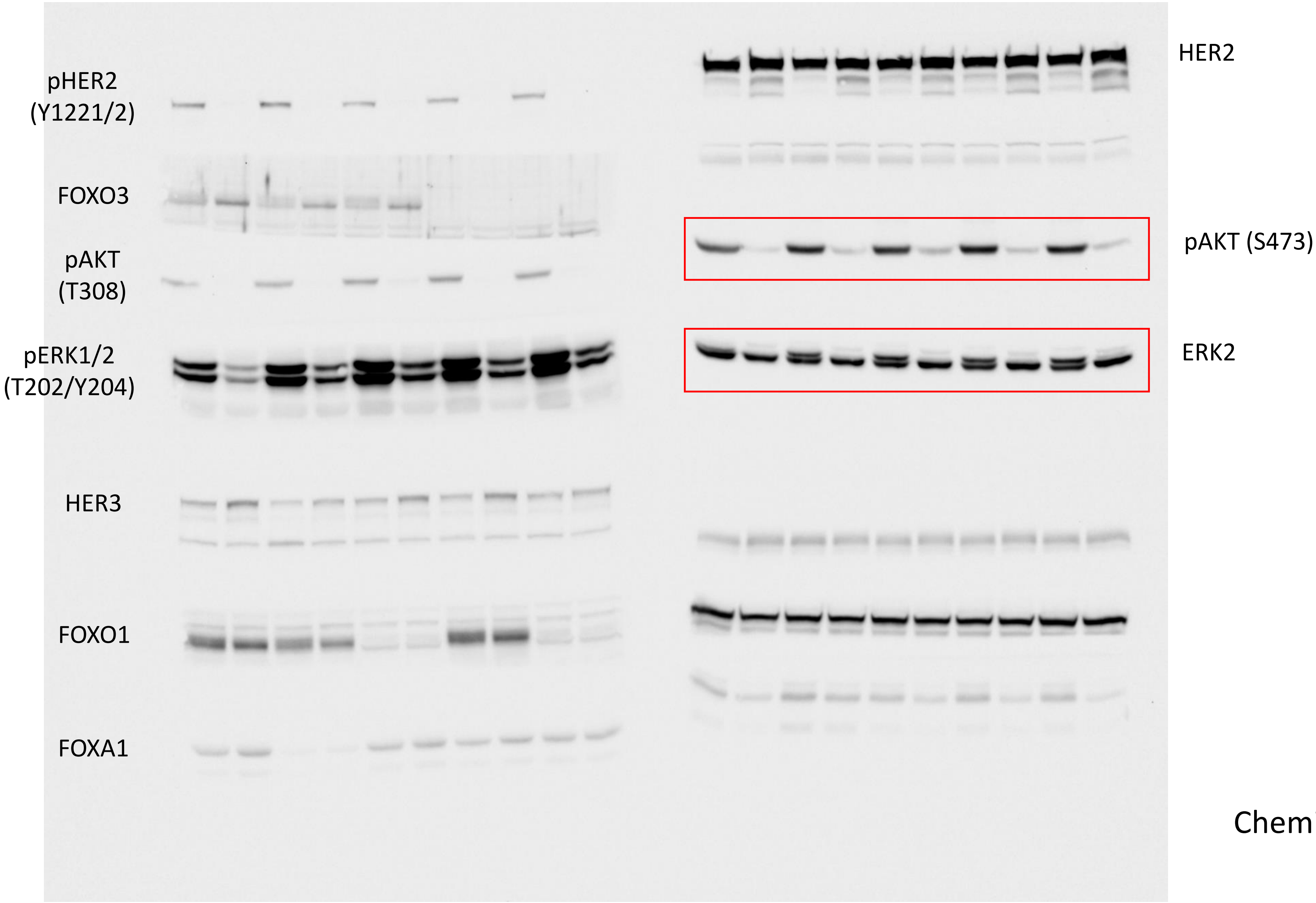

Chemidoc Scans – related to Fig. 3b

Exposure 2

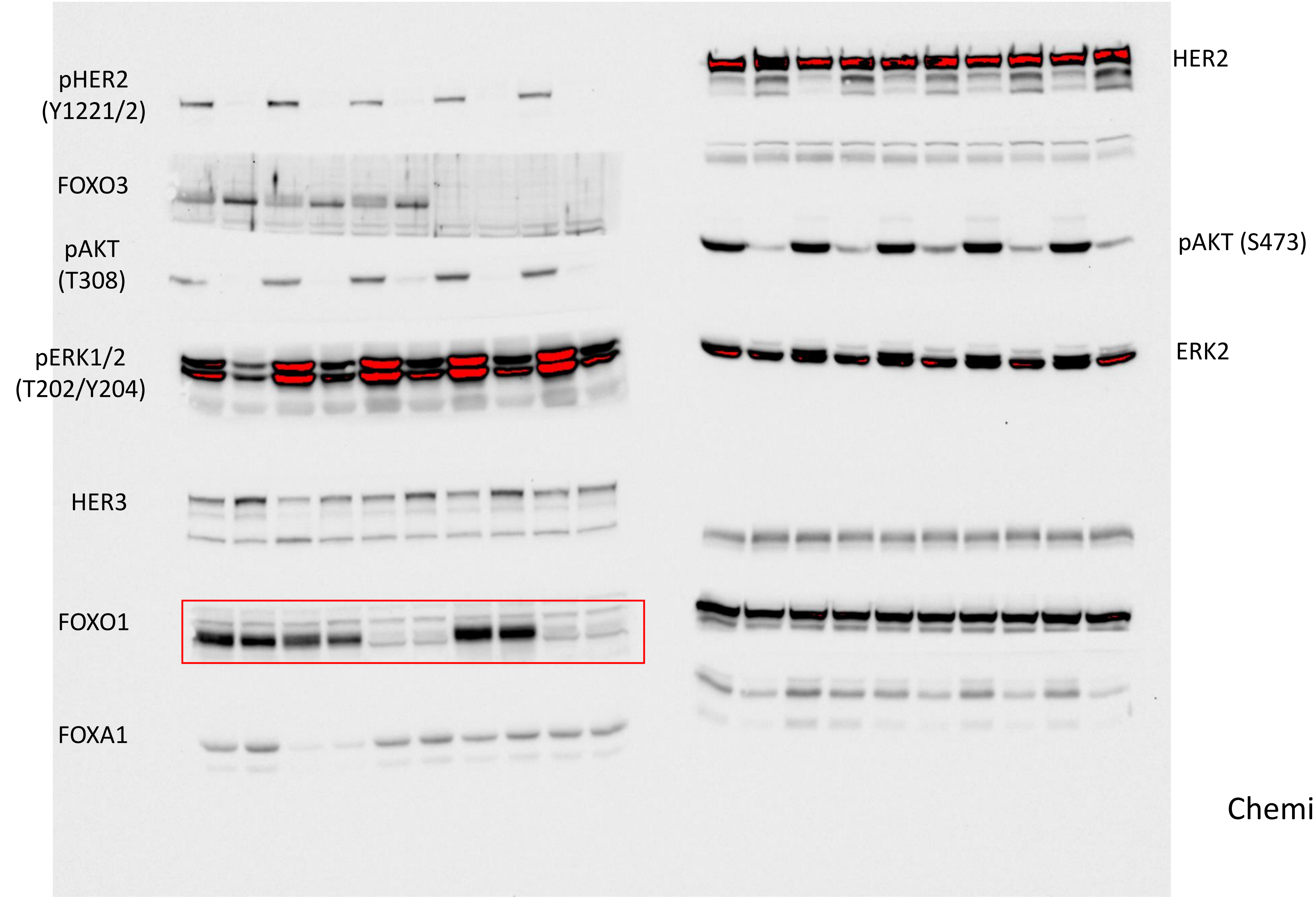

Chemidoc Scans – related to Fig. 3b

Exposure 3

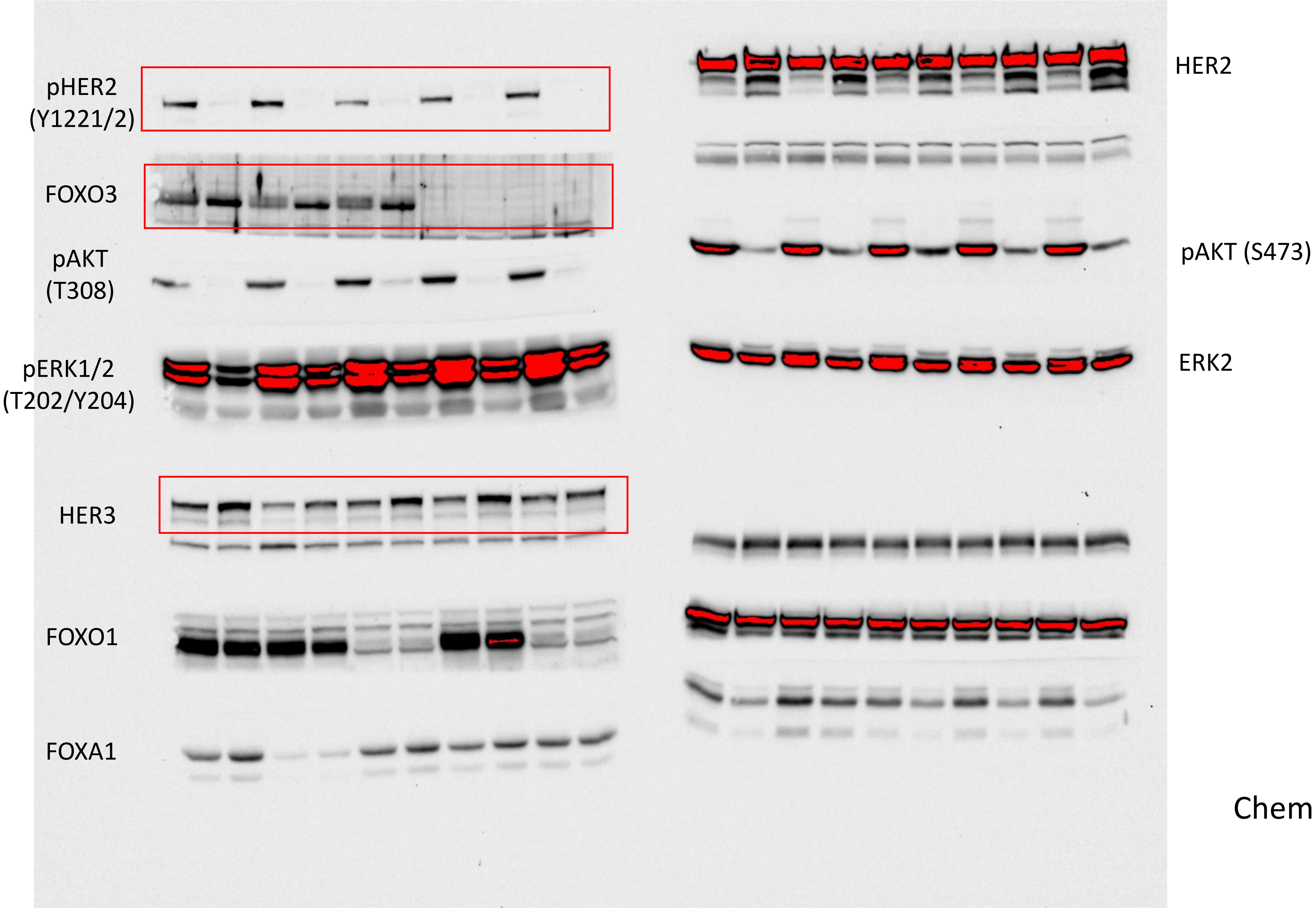

Chemidoc Scans – related to Fig. 3b

Exposure 4

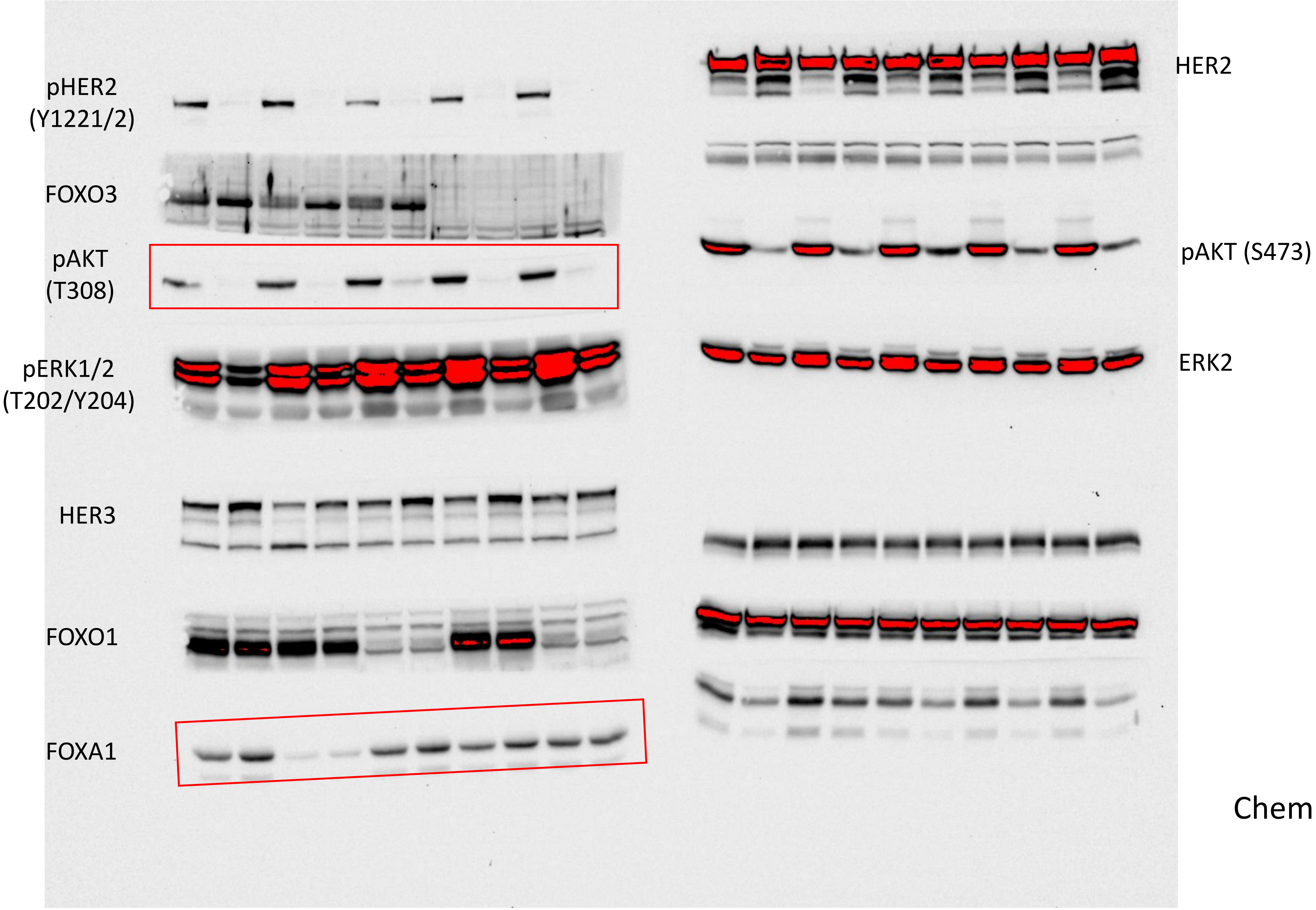

Chemidoc Scans – related to Fig. 3b

Exposure 5

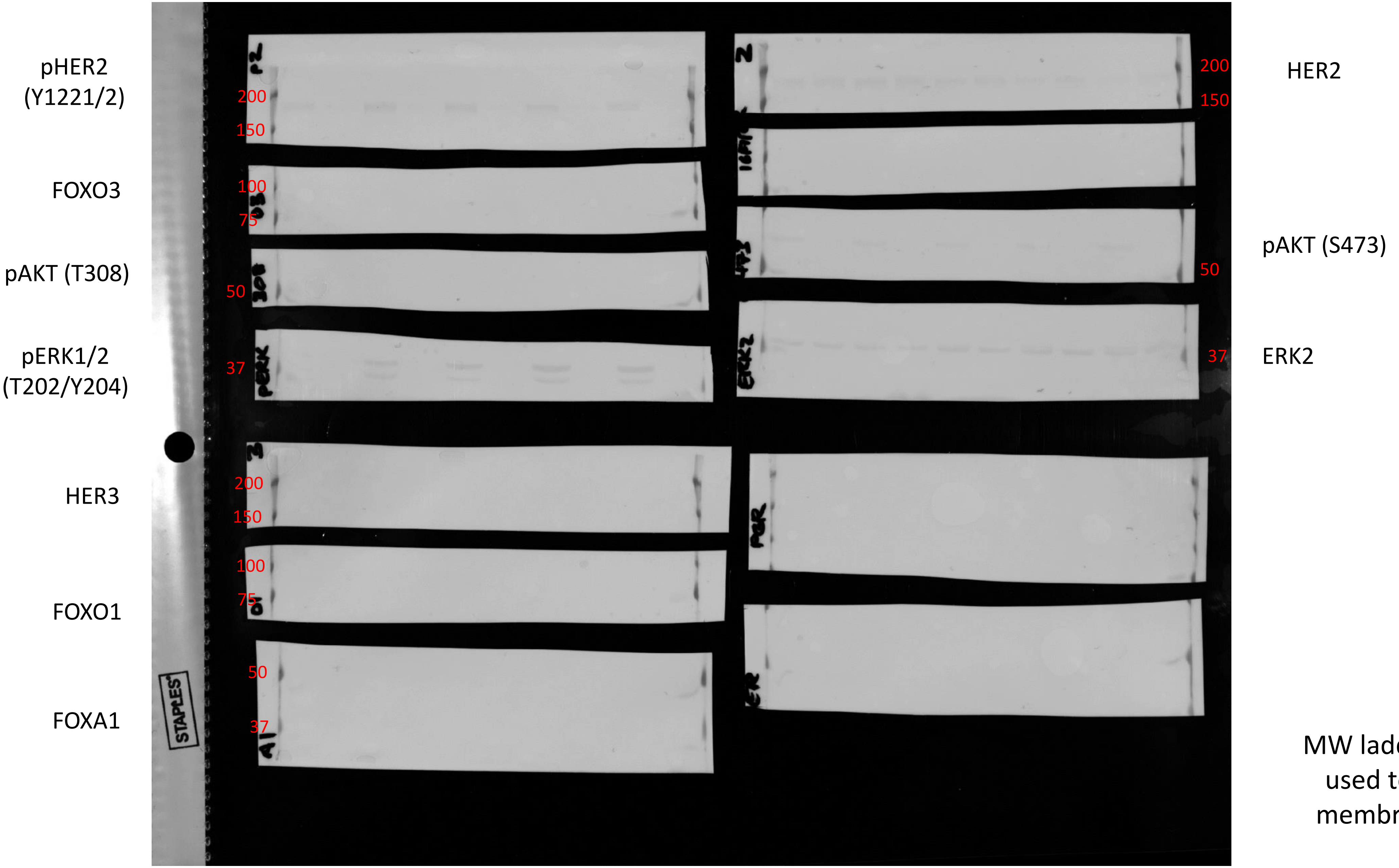

MW ladder marks used to guide membrane cuts

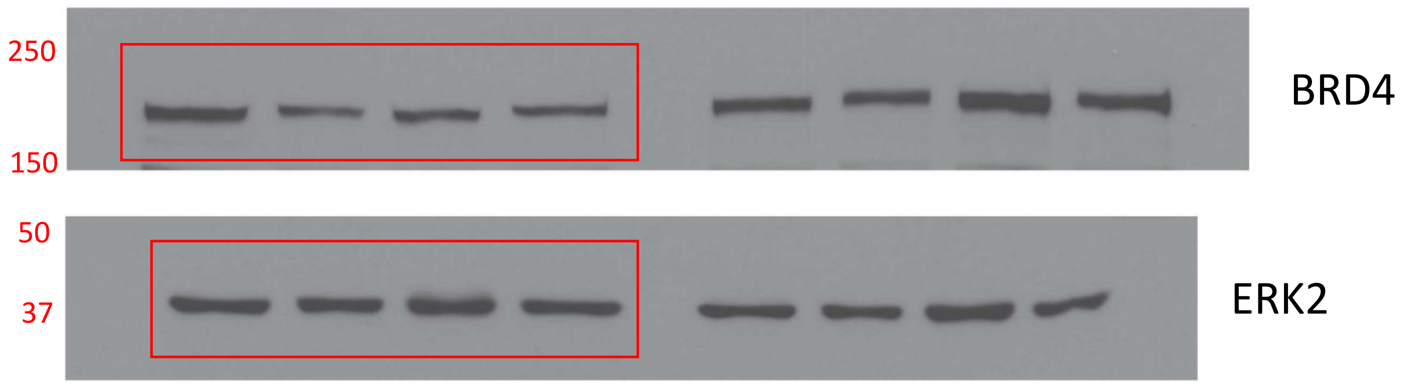

Uncropped Western blot Image Scans – related to Fig. 1f
