# Supplementary material for: FOXA1 and adaptive response determinants to HER2 targeted therapy in TBCRC 036
Source: NPJ Breast Cancer. 2021 May 12;7:51. doi: 10.1038/s41523-021-00258-0 (PMC8115531; doi:10.1038/s41523-021-00258-0)
Supplement: Supplementary file 12 — Reporting Summary [file 41523_2021_258_MOESM12_ESM.pdf]

nature research

Corresponding author(s): Johnson, GL

Last updated by author(s): 01/11/2021

Reporting Summary

Nature Research wishes to improve the reproducibility of the work that we publish. This form provides structure for consistency and transparency in reporting. For further information on Nature Research policies, see our Editorial Policies and the Editorial Policy Checklist.

Statistics

For all statistical analyses, confirm that the following items are present in the figure legend, table legend, main text, or Methods section.

n/a	Confirmed	
☐	☒	The exact sample size (n) for each experimental group/condition, given as a discrete number and unit of measurement
☐	☒	A statement on whether measurements were taken from distinct samples or whether the same sample was measured repeatedly
☐	☒	The statistical test(s) used AND whether they are one- or two-sided<br>*Only common tests should be described solely by name; describe more complex techniques in the Methods section.*
☐	☒	A description of all covariates tested
☐	☒	A description of any assumptions or corrections, such as tests of normality and adjustment for multiple comparisons
☐	☒	A full description of the statistical parameters including central tendency (e.g. means) or other basic estimates (e.g. regression coefficient) AND variation (e.g. standard deviation) or associated estimates of uncertainty (e.g. confidence intervals)
☐	☒	For null hypothesis testing, the test statistic (e.g. F, t, r) with confidence intervals, effect sizes, degrees of freedom and P value noted<br>*Give P values as exact values whenever suitable.*
☒	☐	For Bayesian analysis, information on the choice of priors and Markov chain Monte Carlo settings
☐	☒	For hierarchical and complex designs, identification of the appropriate level for tests and full reporting of outcomes
☒	☐	Estimates of effect sizes (e.g. Cohen's d, Pearson's r), indicating how they were calculated

Our web collection on statistics for biologists contains articles on many of the points above.

Software and code

Policy information about availability of computer code

Data collection	No software was used to collect data in the study.
Data analysis	For RNAseq from cell lines, QC-passed reads were aligned to the human reference genome (hg19) using MapSplice. The alignment profile was determined by Picard Tools v1.64. Aligned reads were sorted and indexed using SAMtools and translated to transcriptome coordinates and filtered for indels, large inserts, and zero mapping quality using UBU v1.0. Transcript abundance estimates for each sample were performed using an expectation–maximization algorithm, RSEM. Expected read counts for genes were used as input for DESeq2 to identify differentially expressed genes. For RNAseq from patient samples, QC-passed reads were aligned to the human reference genome (hg38) using STAR 2.4.2a and reads were translated to transcriptome coordinates using Salmon 0.60. Isoform data were collated to single gene IDs using the R package biomaRt, and abundance estimates were upper quartile normalized using R. Python code generated in the laboratory for ChIPseq analysis is available at GitHub https://github.com/darshansinghunc/chippeakanalysis. For kinome proteomics, Thermo MS raw files were processed using the MaxQuant version 1.5.1.2. The data were searched against a reviewed Uniprot/Swiss-Prot human database. Fixed modification: carbamidomethylation (C) and variable modifications: oxidation (M), phospho (STY), Acetyl (Protein N-term) and deamidation (NQ). Default 1% false discovery rate was used, with unique peptides used for label-free quantification (MaxLFQ). Matching between runs was enabled with 3 min match time window. Kinases were annotated from the data set and those with >1 unique peptide were kept. Using Perseus (1.6.10.50), LFQ intensities were log2 transformed and missing LFQ intensities were replaced with the column (sample) minimum value for comparison of matched pre-treatment and post-treatment samples.

For manuscripts utilizing custom algorithms or software that are central to the research but not yet described in published literature, software must be made available to editors and reviewers. We strongly encourage code deposition in a community repository (e.g. GitHub). See the Nature Research guidelines for submitting code & software for further information.

Data

Policy information about availability of data

All manuscripts must include a data availability statement. This statement should provide the following information, where applicable:

- Accession codes, unique identifiers, or web links for publicly available datasets
- A list of figures that have associated raw data
- A description of any restrictions on data availability

The mass spectrometry proteomics data have been deposited to the ProteomeXchange Consortium via the PRIDE partner repository with the data set identifier PXD021865 (During review, the data may be viewed at http://www.ebi.ac.uk/pride, Username: reviewer_pxd021865@ebi.ac.uk and Password: RdRfDFTV). This data is shown in Figure 5). RNAseq and ChIPseq data shown in Figs. 1-6 is available as part of the GEO SuperSeries GSE160670 (https://www.ncbi.nlm.nih.gov/geo/query/acc.cgi?acc=GSE160670). Due to incomplete permissions from participating institutions, we are unable to upload raw data at this time due to patient privacy concerns for SubSeries GSE161743. Normalized counts have been uploaded to GEO and processed data are also included as supplemental material.

Field-specific reporting

Please select the one below that is the best fit for your research. If you are not sure, read the appropriate sections before making your selection.

☒ Life sciences ☐ Behavioural & social sciences ☐ Ecological, evolutionary & environmental sciences

For a reference copy of the document with all sections, see nature.com/documents/nr-reporting-summary-flat.pdf

Life sciences study design

All studies must disclose on these points even when the disclosure is negative.

Sample size	The clinical trial was designed with an accrual goal of 10 patients per treatment arm but this accrual was not achieved. Final accrual was 7 patients in Arm A, 6 patients in Arm B, 5 patients in Arm C, and 8 patients in Arm D but matched or evaluable biological specimens were not obtained for all patients (see below, 13 patient-matched and 8 unmatched samples were used for molecular analysis). RNAseq and ChIPseq was performed singly with the intent of identifying common enrichment in response to lapatinib treatment. RNAseq following siRNA-mediated knockdown of FOXA1 was performed in biological triplicate.
Data exclusions	For kinome proteomics, samples with >50% imputed log2LFQ intensities were excluded (117-pre (HER2_19) and 112-pre (HER2_11). The post-treatment sample from patient 111 (111-post (HER2_10) was re-run due to poor chromatography and the re-run sample (111-post (HER2_10rerun)) was used for analysis. Patient 103 samples were re-run (103-Rb and 103-Ob) but the original sample files from freshly prepared lysate were used for analysis (103-Ra and 103-Oa (HER2_03 and HER2_04)). Lysate from human HER2+ patient-derived xenografts WHIM8 and WHIM35 were processed and searched in parallel to improve matching between runs in MaxLFQ. The log2 LFQ difference for matched patient pre- and post-treatment samples was compared.
Replication	Enrichment by ChIPseq and expression of proximal genes in response to lapatinib identified the common FOXA1-binding motif. Disruption of enhancers and effect on cell proliferation was consistently observed in response to FOXA1 depletion. Immunoblotting and cell proliferation assays were performed at least twice and representative data are shown.
Randomization	Samples were randomly assigned for siRNA or drug treatment of cell lines grown in parallel. Patients were randomized into treatment arms of the clinical trial.
Blinding	Investigators were not blinded to group allocation and blinding was not relevant as comparisons were normalized to internal controls (e.g. non-targeting siRNA, DMSO treatment, or input DNA). Investigators were not blinded to analysis of patient samples so same-patient comparisons (pre-treatment vs post-treatment) could be made from the resultant raw data files.

Reporting for specific materials, systems and methods

We require information from authors about some types of materials, experimental systems and methods used in many studies. Here, indicate whether each material, system or method listed is relevant to your study. If you are not sure if a list item applies to your research, read the appropriate section before selecting a response.

Materials & experimental systems

n/a	Involved in the study	
☐	☒	Antibodies
☐	☒	Eukaryotic cell lines
☒	☐	Palaeontology and archaeology
☒	☐	Animals and other organisms
☐	☒	Human research participants
☐	☒	Clinical data
☒	☐	Dual use research of concern

Methods

n/a	Involved in the study	
☐	☒	ChIP-seq
☒	☐	Flow cytometry
☒	☐	MRI-based neuroimaging

Antibodies

Antibodies used	FOXA1 (abcam #ab5089), BRD4 (Bethyl Laboratories #A301-985A100), Histone H3K27ac (Active Motif #39133), MED1 (Bethyl Laboratories #A300-793A100), Phospho-HER2/ErbB2 (Tyr1221/1222) (6B12) Rabbit mAb (Cell Signaling #2243), HER2/ErbB2 (29D8) Rabbit mAb (Cell Signaling #2165), FoxO1 (C29H4) Rabbit mAb (Cell Signaling #2880), FoxO3a (75D8) Rabbit mAb (Cell Signaling #2497), HER3/ErbB3 (D22C5) XP® Rabbit mAb (Cell Signaling #12708), Phospho-Akt (Ser473) (D9E) XP® Rabbit mAb (Cell Signaling #4060), Phospho-Akt (Thr308) (D25E6) XP® Rabbit mAb (Cell Signaling #13038), Phospho-p44/42 MAPK (Erk1/2) (Thr202/Tyr204) (D13.14.4E) XP® Rabbit mAb (Cell Signaling #4370), ERK2 Antibody (C-14): Santa Cruz sc-154
Validation	Validation for given applications provided by source companies: Santa Cruz (scbt.com), Cell Signaling Technology (cellsignal.com), Abcam (abcam.com), Bethyl Laboratories (bethyl.com), Active Motif (activemotif.com) and our own previous usage for western blot and ChIPseq.

Eukaryotic cell lines

Policy information about cell lines

Cell line source(s)	UNC Cell Culture Facility (SKBR-3) and MC Hung, MD Anderson Cancer Center, TX (BT474.m1).
Authentication	Cell lines were not further authenticated after receipt from source.
Mycoplasma contamination	Cells tested negative for mycoplasma contamination by DAPI staining.
Commonly misidentified lines (See ICLAC register)	No commonly misidentified cell lines were used.

Human research participants

Policy information about studies involving human research participants

Population characteristics	The LCCC1214 / TBCRC 036 window trial, "Defining the HER2 Positive (+) Breast Cancer Kinome Response to Trastuzumab, Pertuzumab, Combination Trastuzumab + Pertuzumab, or Combination Trastuzumab + Lapatinib" is registered under the ClinicalTrials.gov identifier NCT01875666 (date of registration, 6/13/2014). GlaxoSmithKline generously provided lapatinib (Tykerb™) and Genentech, Inc. generously provided trastuzumab (Herceptin™) and pertuzumab (Perjeta™) for the study. Eligible women included those with newly diagnosed Stage I-IV HER2+ breast cancer scheduled to undergo definitive surgery (either lumpectomy or mastectomy). Stage I-IIIc patients could not be candidates for a therapeutic neoadjuvant treatment. Histological confirmation of HER2+ status was determined by IHC 3+ or by fluorescence in situ hybridization (FISH), clinical assays on either primary or metastatic tumor. Study subjects provided informed written consent that included details of the nontherapeutic nature of the trial, and the study was approved by the UNC Office of Human Research Ethics and conducted in accordance with the Declaration of Helsinki. Twenty-six patients were enrolled at 4 institutions (UAB, UNC, Dana Farber, MD Anderson) between 10/31/2013 and 12/6/2016 and randomized to a treatment arm (7A, 6B, 5C, 8D). Some patients either declined a required study procedure after enrollment or were found ineligible (e.g. ineligible based on pretreatment laboratory values), some of whom had research biopsies already collected on an institutional tissue banking protocol so could contribute pre-treatment samples for analysis but could not contribute post-treatment samples. No patient had a treatment-related adverse event during the course of study treatment or follow-up. The study was closed for completion of enrollment for this purely correlative trial. For 13 of these patients, matched pre-treatment and post-treatment samples were collected while for 8 additional patients, only a single pre-treatment or post-treatment sample of sufficient quality was available.
Recruitment	Eligible women included those with newly diagnosed Stage I-IV HER2+ breast cancer scheduled to undergo definitive surgery (either lumpectomy or mastectomy). Stage I-IIIc patients could not be candidates for a therapeutic neoadjuvant treatment. Study subjects provided informed written consent that included details of the nontherapeutic nature of the trial.
Ethics oversight	The study was approved by the UNC Office of Human Research Ethics, IRB# 13-1826.

Note that full information on the approval of the study protocol must also be provided in the manuscript.

Clinical data

Policy information about clinical studies

All manuscripts should comply with the ICMJE guidelines for publication of clinical research and a completed CONSORT checklist must be included with all submissions.

Clinical trial registration	ClinicalTrials.gov Identifier: NCT01875666
Study protocol	Available as supplemental material.
Data collection	Twenty-six patients were enrolled at 4 institutions (UAB, UNC, Dana Farber, MD Anderson) between 10/31/2013 and 12/6/2016 and randomized to a treatment arm (7A, 6B, 5C, 8D). Some patients either declined a required study procedure after enrollment or were found ineligible (e.g. ineligible based on pretreatment laboratory values), some of whom had research biopsies already collected on an institutional tissue banking protocol so could contribute pre-treatment samples for analysis but could not contribute post-treatment samples. No patient had a treatment-related adverse event during the course of study treatment or follow-up. The study was closed for completion of enrollment for this purely correlative trial. For 13 of these patients, matched pre-treatment and post-

	treatment samples were collected while for 8 additional patients, only a single pre-treatment or post-treatment sample of sufficient quality was available.
Outcomes	Not applicable, clinical outcome not included. Only molecular analysis was performed (RNA sequencing and kinase enrichement proteomics).

ChIP-seq

Data deposition

☒ Confirm that both raw and final processed data have been deposited in a public database such as GEO.

☒ Confirm that you have deposited or provided access to graph files (e.g. BED files) for the called peaks.

Data access links<br>*May remain private before publication.*	GSE160667, part of GEO SuperSeries GSE160670 (https://www.ncbi.nlm.nih.gov/geo/query/acc.cgi?acc=GSE160670).
Files in database submission	BT474m1_H3K27_DMSO_S5_R1_001.fastq.gz<br>BT474m1_H3K27_JQ1_S7_R1_001.fastq.gz<br>BT474m1_H3K27_lapatJQ1_S8_R1_001.fastq.gz<br>BT474m1_H3K27_lapat_S6_R1_001.fastq.gz<br>BT474m1_input_DMSO_S9_R1_001.fastq.gz<br>SKBR3_input_DMSO_S11_R1_001.fastq.gz<br>SKBR3_MED1_DMSO_S7_R1_001.fastq.gz<br>SKBR3_MED1_JQ1_S9_R1_001.fastq.gz<br>SKBR3_MED1_lapatJQ1_S10_R1_001.fastq.gz<br>SKBR3_MED1_lapat_S8_R1_001.fastq.gz<br>TJS150525_SKBR3_BRD4_300nMJQ1_24h_S3_sorted.fastq.gz<br>TJS150525_SKBR3_BRD4_300nMlapatinib_24h_S2_sorted.fastq.gz<br>TJS150525_SKBR3_BRD4_300nMlapatinib_300nMJQ1_24h_S4_sorted.fastq.gz<br>TJS150525_SKBR3_BRD4_DMSO_24h_S1_sorted.fastq.gz<br>TJS150525_SKBR3_H3K27ac_300nMJQ1_24h_S7_sorted.fastq.gz<br>TJS150525_SKBR3_H3K27ac_300nMlapatinib_24h_S6_sorted.fastq.gz<br>TJS150525_SKBR3_H3K27ac_300nMlapatinib_300nMJQ1_24h_S8_sorted.fastq.gz<br>TJS150525_SKBR3_H3K27ac_DMSO_24h_S5_sorted.fastq.gz<br>TJS150830_SKBR3_FOXA1_300nMJQ1_24h_S9_sorted.fastq.gz<br>TJS150830_SKBR3_FOXA1_300nMlapatinib_24h_S8_sorted.fastq.gz<br>TJS150830_SKBR3_FOXA1_300nMlapatinib_300nMJQ1_24h_S10_sorted.fastq.gz<br>TJS150830_SKBR3_FOXA1_DMSO_24h_S7_sorted.fastq.gz<br>TJS150830_SKBR3_input_DMSO_24h_S11_sorted.fastq.gz<br>TJS160421_BT474m1_BRD4_300nMJQ1_24h_S3_R1_001.fastq.gz<br>TJS160421_BT474m1_BRD4_300nMlapatinib_24h_S2_R1_001.fastq.gz<br>TJS160421_BT474m1_BRD4_300nMlapatinib_300nMJQ1_24h_S4_R1_001.fastq.gz<br>TJS160421_BT474m1_BRD4_DMSO_24h_S1_R1_001.fastq.gz<br>TJS160421_BT474m1_input_DMSO_24h_S9_R1_001.fastq.gz<br>TJS160421_BT474m1_MED1_300nMJQ1_24h_S7_R1_001.fastq.gz<br>TJS160421_BT474m1_MED1_300nMlapatinib_24h_S6_R1_001.fastq.gz<br>TJS160421_BT474m1_MED1_300nMlapatinib_300nMJQ1_24h_S8_R1_001.fastq.gz<br>TJS160421_BT474m1_MED1_DMSO_24h_S5_R1_001.fastq.gz<br>TJS160613_SKBR3_BRD4_siFOXA1_24h_300nMlapatinib_24h_S4_R1_001.fastq.gz<br>TJS160613_SKBR3_BRD4_siFOXA1_24h_DMSO_24h_S3_R1_001.fastq.gz<br>TJS160613_SKBR3_BRD4_siNT_24h_300nMlapatinib_24h_S2_R1_001.fastq.gz<br>TJS160613_SKBR3_BRD4_siNT_24h_DMSO_24h_S1_R1_001.fastq.gz<br>TJS160613_SKBR3_input_siFOXA1_24h_DMSO_24h_S10_R1_001.fastq.gz<br>TJS160613_SKBR3_input_siNT_24h_DMSO_24h_S9_R1_001.fastq.gz<br>TJS160613_SKBR3_MED1_siFOXA1_24h_300nMlapatinib_24h_S8_R1_001.fastq.gz<br>TJS160613_SKBR3_MED1_siFOXA1_24h_DMSO_24h_S7_R1_001.fastq.gz<br>TJS160613_SKBR3_MED1_siNT_24h_300nMlapatinib_24h_S6_R1_001.fastq.gz<br>TJS160613_SKBR3_MED1_siNT_24h_DMSO_24h_S5_R1_001.fastq.gz<br>SKBR3_BRD4_DMSO.bw<br>SKBR3_BRD4_JQ1.bw<br>SKBR3_BRD4_lapat.bw<br>SKBR3_BRD4_lapatJQ1.bw<br>SKBR3_H3K27ac_DMSO.bw<br>SKBR3_H3K27ac_JQ1.bw<br>SKBR3_H3K27ac_lapat.bw<br>SKBR3_H3K27ac_lapatJQ1.bw<br>SKBR3_input_DMSO.bw<br>SKBR3_MED1_DMSO.bw<br>SKBR3_MED1_JQ1.bw<br>SKBR3_MED1_lapat.bw<br>SKBR3_MED1_lapatJQ1.bw<br>TJS150723_BT474m1_H3K27ac_300nMJQ1_24h_S7_sorted.bw<br>TJS150723_BT474m1_H3K27ac_300nMlapatinib_24h_S6_sorted.bw<br>TJS150723_BT474m1_H3K27ac_300nMlapatinib_300nMJQ1_24h_S8_sorted.bw

TJS150723_BT474m1_H3K27ac_DMSO_24h_S5_sorted.bw
TJS150723_BT474m1_input_DMSO_24h_S9_sorted.bw
TJS150830_SKBR3_FOXA1_300nMJQ1_24h_S9_sorted.bw
TJS150830_SKBR3_FOXA1_300nMlapatinib_24h_S8_sorted.bw
TJS150830_SKBR3_FOXA1_300nMlapatinib_300nMJQ1_24h_S10_sorted.bw
TJS150830_SKBR3_FOXA1_DMSO_24h_S7_sorted.bw
TJS150830_SKBR3_input_DMSO_24h_S11_sorted.bw
TJS160421_BT474m1_BRD4_300nMJQ1_24h_S3_R1_sorted.bw
TJS160421_BT474m1_BRD4_300nMlapatinib_24h_S2_R1_sorted.bw
TJS160421_BT474m1_BRD4_300nMlapatinib_300nMJQ1_24h_S4_R1_sorted.bw
TJS160421_BT474m1_BRD4_DMSO_24h_S1_R1_sorted.bw
TJS160421_BT474m1_input_DMSO_24h_S9_R1_sorted.bw
TJS160421_BT474m1_MED1_300nMJQ1_24h_S7_R1_sorted.bw
TJS160421_BT474m1_MED1_300nMlapatinib_24h_S6_R1_sorted.bw
TJS160421_BT474m1_MED1_300nMlapatinib_300nMJQ1_24h_S8_R1_sorted.bw
TJS160421_BT474m1_MED1_DMSO_24h_S5_R1_sorted.bw
TJS160613_SKBR3_BRD4_siFOXA1_24h_300nMlapatinib_24h_S4_R1_sorted.bw
TJS160613_SKBR3_BRD4_siFOXA1_24h_DMSO_24h_S3_R1_sorted.bw
TJS160613_SKBR3_BRD4_siNT_24h_300nMlapatinib_24h_S2_R1_sorted.bw
TJS160613_SKBR3_BRD4_siNT_24h_DMSO_24h_S1_R1_sorted.bw
TJS160613_SKBR3_input_siFOXA1_24h_DMSO_24h_S10_R1_sorted.bw
TJS160613_SKBR3_input_siNT_24h_DMSO_24h_S9_R1_sorted.bw
TJS160613_SKBR3_MED1_siFOXA1_24h_300nMlapatinib_24h_S8_R1_sorted.bw
TJS160613_SKBR3_MED1_siFOXA1_24h_DMSO_24h_S7_R1_sorted.bw
TJS160613_SKBR3_MED1_siNT_24h_300nMlapatinib_24h_S6_R1_sorted.bw
TJS160613_SKBR3_MED1_siNT_24h_DMSO_24h_S5_R1_sorted.bw
SKBR-3-BRD4-MED1-H3K27Ac-FOXA1-Lapatinib-JQ1.xlsx
BT474m1-BRD4-MED1-H3K27Ac-Lapatinib-JQ1.xlsx
SKBR-3-BRD4-MED1-siNontargeting-siFOXA1-Lapatinib.xlsx

Genome browser session
(e.g. UCSC)

N/A

Methodology

Replicates

ChIPseq experiments were performed once with emphasis on common marker enrichment to identify putative enhancer regions.

Sequencing depth

All ChIP sequencing was performed as 12- or 13-plex experiments, single-end 75 bp reads, using an Illumina NextSeq 500 High Output Kit (400 million reads per run) resulting in 35-40 million reads per sample and 22-25 million uniquely mapped reads per sample.

Antibodies

FOXA1 (abcam #ab5089), BRD4 (Bethyl Laboratories #A301-985A100), Histone H3K27ac (Active Motif #39133), MED1 (Bethyl Laboratories #A300-793A100).

Peak calling parameters

Reads were aligned to hg19 using Bowtie v1.1.2 with parameters -v 2 -m 1. The following processing steps were performed to generate peakclassification files: Calculating read density: The density of reads in each region was normalized to the total number of million mapped reads producing read density in units of reads per million mapped reads (rpm). The read density of the input chromatin was subtracted from the ChIP read density for normalization. Peak calling: We used a combination of two algorithms, MACS v1.4.2 and HMCan v1.21, to define enriched regions. We used HMCan to call peaks in regions of high CNV, defined as regions of size > 50 kb where no MACS peaks are called, and have read coverage that is >3 times the average read coverage. Peaks within 12.5 kb of each other were stitched as described in Loven et al. 2013 Cell 153. We used default settings for MACS, and HMCan was run with narrow peak calling configuration file with no blacklisted regions. Peak genic classification: A peak region was classified on the basis of its location with respect to GRCh37/hg19 gene annotations. If the region was +/- 5 kb of any transcription start site, it was classified as a promoter peak. If it was within -5kb to -200kb of any transcriptional start site, it was classified as a 5' enhancer peak. If the peak overlapped the gene boundary, and was not classified as a promoter or enhancer peak, it was classified as either a genebody_exon or genebody_intron peak. If the peak resided within 0 to +200kb from the 3'-most exon, and did not fulfill the criteria for the above classifications, it was classified as a 3' enhancer. All remaining peaks were designated as "other". For comparative analysis, the treatment datasets were clubbed together in a project. A common set of regions was needed to compare ChIPseq enrichment upon drug treatment or siRNA treatment, we thus defined union peaks for a given project: a collection of peak regions of all the treatment datasets in a project, with adjacent peaks merged.

Data quality

FastQC was run on all samples. to ensure quality. Peak calls are available as Supplemental Tables S1-S3 and available via GEO.

Software

Python code generated in the laboratory for ChIPseq analysis is available at GitHub https://github.com/darshansinghunc/chippeakanalysis and requires Python 2.7, MACS 1.4.2, HMCan 1.21, and samtools 1.2.
